# Effects of protein G-quadruplex interactions on phase transitions and protein aggregation

**DOI:** 10.1101/2023.09.21.558871

**Published:** 2023-09-21

**Authors:** Bikash R. Sahoo, Vojč Kocman, Nathan Clark, Nikhil Myers, Xiexiong Deng, Ee L. Wong, Harry J. Yang, Anita Kotar, Bryan B. Guzman, Daniel Dominguez, Janez Plavec, James C.A. Bardwell

**Affiliations:** 1 Howard Hughes Medical Institute; 2 Department of Molecular, Cellular and Developmental Biology, University of Michigan, Ann Arbor, MI, USA; 3 National Institute of Chemistry, Ljubljana, Slovenia; 4 Department of Pharmacology, UNC Chapel Hill, USA

## Abstract

We show that human protein Znf706 interacts specifically with stable, non-canonical nucleic acid structures known as G-quadruplexes. Znf706, though only 76 residues long, is comprised of two distinct domains, one disordered and one ordered. The disordered domain is homologous to the SERF family of proteins and acts to accelerate amyloid formation. The ordered domain contains a C2H2 type zinc-finger. We show that Znf706 not only accelerates amyloid formation but also accelerates amorphous protein aggregation. We find that Znf706 binds preferentially to parallel G-quadruplexes with low micromolar affinity, primarily using its N-terminus, whose dynamics are constrained upon interaction. G-quadruplexes are potent anti-aggregation agents, and their binding to Znf706 suppresses Znf706’s ability to accelerate protein aggregation and fibril formation. Znf706 in conjunction with G-quadruplexes thus may play a role in regulating protein folding. Depletion of Znf706 specifically impacts mRNA abundance of genes that contain high G-quadruplex density, implying that Znf706 may also serve as a G-quadruplex specific regulator. Our studies give insights into how proteins and G-quadruplexes interact, how these interactions affect both partners, lead to liquid-liquid phase transitions, and lead to the modulation of protein aggregation and cellular mRNA levels.

## Introduction

A surprisingly sizable percentage of proteins, including those in broadly conserved families, still lack clearly defined functions. This is particularly an issue for proteins containing regions of disorder^1-4^. The sequence properties driving disorder in proteins and disorder’s overall importance in protein function have been the subject of innumerable reviews^5^. However, the inherent difficulty in studying disorder makes it particularly challenging to clearly understand in detail how disorder contributes to protein function. While some details are emerging about how disordered proteins interact with each other^6^, there is limited availability of high-resolution structural information for disordered proteins in isolation. This information is a prerequisite for understanding how and why disordered proteins interact with their binding partners.

We have decided to study one such family of partially disordered proteins, namely the 4F5 family (Pfam: PF04419). The founding member of this family, Small ERDK-rich Factor (SERF), was discovered by its ability to accelerate amyloid aggregation in both a *C. elegans* model of polyglutamine aggregation disease and in vitro for α-synuclein which is associated with Parkinson’s disease, and β-amyloid peptides which are associated with Alzheimer’s disease^7^. SERF proteins form fuzzy complexes with amyloid precursors which accelerate amyloid nucleation^8^. These proteins are characterized as having high net charge, a high degree of conservation, and partial structural disorder^7-10^. Recently, it has been found that SERF proteins bind to nucleic acids in vitro and localize to nucleic acid-rich membrane-less compartments such as nucleoli in vivo, yet the SERF protein’s biological roles in normal cell physiology have remained elusive^11,12^.

Around 40% of 4F5 family proteins are characterized by an N-terminal SERF-homologous domain and a C-terminal single C2H2 type zinc-finger domain^11^. We have focused on one such protein, an ~8.5 kDa human protein called Znf706. Most commonly, zinc-finger proteins interact with nucleic acid structures act to regulate gene expression using multiple zinc-finger domains^13^, conspicuously, Znf706 has only a single zinc-finger. Though this protein has family members in a wide variety of eukaryotic organisms, little is known as to it or its family members’ functions^14,15^.

Presented here is evidence that Znf706 specifically interacts with G-quadruplexes. G-quadruplexes are non-canonical, guanine rich nucleic acid structures that form G-tetrads, planar Hoogsteen base-paired arrangements of four guanine residues stabilized by monovalent cations (K^+^/Na^+^). These G-tetrads can stack over one another through π-π integration to form compact and ordered structures^16^. G-quadruplex forming sequences have been detected in telomeres and in the untranslated regions of many genes^17^. They are thought to regulate telomere function and cellular processes such as gene regulation and mRNA stability, particularly under stress conditions^16,18-20^. Several proteins are known to regulate G-quadruplex function. Helicases, such as DHX36, DDX5, FANCJ, POT1, RPA, are capable of unfolding G-quadruplexes^21-25^, and nucleolin, p53, DNMT1, along with several zinc-finger proteins, including Sp1 and MAZ, are known to bind to and stabilize G-quadruplexes^26^. G-quadruplex structure and polymorphism have been extensively studied, and several high-resolution structures of G-quadruplexes complexed with proteins including DHX36 helicase^22^, yeast telomeric protein Rap1^27^, and telomere end binding protein in *Oxytricha nova*^28^ have been reported. However, the structural characteristics of G-quadruplexes that allow for protein recognition and knowledge of the dynamical changes that occur in both the G-quadruplex and protein partners upon binding are unclear. Also, unclear is how binding affects the activity of both partners. This is unfortunate given the emerging links between G-quadruplexes and certain protein folding diseases^29^. Examples of this are G-quadruplexes having been found in amyloid aggregates and the discovery that several amyloidogenic proteins bind G-quadruplexes^29,30^. A variety of biophysical tools have enabled us to gain new insight into G-quadruplex and its protein partners. Utilizing them, we have developed a detailed characterization of the nature of protein and G-quadruplex interactions, identified how they participate in liquid-liquid phase transitions, determined the structural changes that occur in both binding partners upon interaction, and hypothesized how these interactions may affect G-quadruplex and Znf706’s opposing activities in controlling protein aggregation and in vivo mRNA levels.

## Results

### Structure of Znf706

**Fig. 1 ∣:**
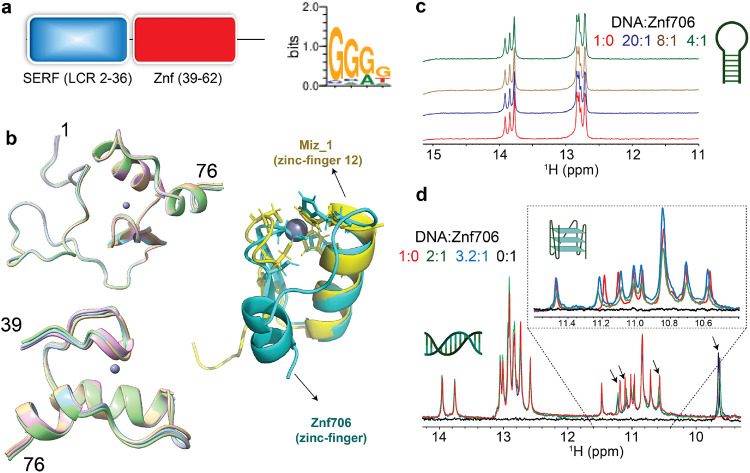
Znf706 is partially disordered and prefers to bind G-quadruplexes. **(a)** Schematic diagram showing that Znf706’s domain organization includes a conserved N-terminal low-complexity domain, colored in blue (residues 2-36) that is homologous to the 4F5/SERF family and a single C2H2 type zinc-finger domain that is shown in red (residues 39-62). The DNA binding specificity of this zinc finger in Znf706 was predicted using the interactive PWM predictor^35^ to be GGGG, with the first residues of the motif more favored to be G than the latter ones. **(b)** De novo structures of full-length (1-76) and Znf706 (39-76) generated by Chemical-Shift-ROSETTA using Cα, Cβ, CO, N, Hα and NH NMR chemical shifts. Superimposed zinc coordinated structures of CS-ROSETTA Znf706 models are generated using HADDOCK. Cartoon structures of the CS-ROSETTA model of the human Znf706 zinc-finger domain in cyan superimposed with the solution NMR model structure of C2H2 type Miz-1 (yellow) zinc finger domain-12 (PDB ID: 7MC3). **(c)** 1D ^1^H NMR spectra showing signals of imino protons involved in Watson-Crick base pairs, with no significant change in the chemical shift observed in the presence of Znf706 indicating no interactions between a G-rich (GGA) hairpin DNA (200 μM) and increasing concentrations of Znf706 at the indicated molar excesses of DNA relative to Znf706. **(d)** Competitive NMR titration measurements probing the binding specificity of Znf706 to G-quadruplexes and duplex containing the exact same sequence of Bcl2SH and its complementary strand as listed in Table 1. The arrows indicate peaks showing substantial chemical shift change upon Znf706 binding to the Bcl2SH G-quadruplex and duplex mixture. Inset shows a zoomed image of the region of the Bcl2SH G-quadruplex imino protons (~10.4-11.5 ppm) that show chemical shift changes. No substantial chemical shifts were observed in the Watson-Crick base pair regions (~12.5-14 ppm) indicating that Znf706 binds exclusively to the G-quadruplex structures of the Bcl2SH sequence. All 1D NMR samples are prepared in 20 mM phosphate buffer containing100 mM KCl and 7.5% D_2_O (pH 7.4).

Though the human Znf706 protein is very small, only 76 amino acids in length, it is predicted using SMART^31^ and IUPred2A^32^ to possess two distinct domains: a disordered, low complexity, N-terminal domain homologous to the 4F5 (SERF) family of proteins^11^ (Figs. 1a and S1a-c), and an ordered C-terminal domain homologous to C2H2 zinc fingers. The SERF homologous domain spans the first 36 residues and the zinc finger domain spans residues 39-62. SERF family proteins were initially characterized by their ability to accelerate amyloid aggregation and later shown to bind nucleic acids^7,8,33^. How these two functions may be connected remains unclear. We decided to attempt to get experimental structural information for Znf706, its small size and solubility made it a good candidate for NMR based approaches. Fortunately, the ^15^N/^1^H heteronuclear correlation spectra of Znf706 are well resolved and allowed the assignment of 57/70 of the non-proline amide peaks (Fig. S1d) observed for zinc-bound Znf706 (Fig. S1e). We were also able to assign the N, Cα, Cβ, NH, and CO backbone chemical shifts for Znf706’s structure determination. The de novo structure we obtained using CS-ROSETTA^34^ from the backbone NMR chemical shifts is shown in Fig. 1b. This structure confirmed predictions that the N-terminal domain (residue 1-36) of Znf706 is dynamic and disordered, and that the C-terminus (residues 39-74) is a folded zinc-finger domain. This zinc finger (Fig. 1b) is characterized by two short anti-parallel β-strands (residues 39-41 and 45-48), two α-helices (residues 51-61 and 69-74) and closely resembles the structure of the C2H2 zinc fingers such as the zinc-finger domain-12 present in Miz-1 (PDB ID: 7MC3) (Fig. 1b).

### Znf706 preferentially binds to G-quadruplexes

To help identify Znf706’s binding preference, we pursued the possibility that Zn706, like the vast majority of C2H2 zinc fingers, interacts with nucleic acids. There is such extensive knowledge about the nucleic acid binding specificity of zinc finger proteins that one can predict their binding specificity from their amino acid sequence alone. We decided to use an interactive position weight matrix tool, the PWM predictor^35^, that utilizes the relationship between protein sequence and nucleic acid binding specificity of zinc fingers. First, we validated the claim made by the writers of this program that it can successfully classify proteins containing known zinc-fingers according to their DNA binding motifs, independent of whether they are G-rich or A and T rich (Fig. S2)^35^. We then inputted the amino acid sequence of Znf706 into the PWM predictor and it gave a binding specificity for Znf706 of GGGG (Fig. 1a). Fluorescence anisotropy showed that polyG DNA binds to Znf706, while polyA, polyT, polyC, and polyN showed no detectable binding (Fig. S3a). PolyG forms G-quadruplexes^36^, so we investigated if Znf706 binds to other known G-quadruplexes. We evaluated sequences that were G-rich but not predicted to form G-quadruplexes (Fig. S3b) nor did so when tested experimentally by fluorescent dyes N-methylmesoporphyrin IX and thioflavin T (ThT) known to bind G-quadruplexes (Table 1 and Figs. S3b and S4). All the oligonucleotides known to form G-quadruplexes when tested showed interaction with Znf706, while all the G-rich sequences we tested known not to form G-quadruplexes failed to bind Znf706 (Fig. S3).

To distinguish between G-quadruplex and duplex binding, we utilized the NMR spectral properties of G-quad-ruplexes. G-quadruplexes have a characteristic chemical shift region around 10.5 and 11.5 ppm where signals resonate from the G-tetrad forming guanine imino protons that are hydrogen bonded in the Hoogsteen geometry. These signals serve as a diagnostic G-quadruplex fingerprint since they are well separated from the imino proton’s signals, stabilized by Watson-Crick bonds, that resonate between 12.5 and 14 ppm. When added to G-rich (GGA or GGGG) hairpin duplex structures, Znf706 was found to be unable to induce changes in proton chemical shifts. This indicates that Znf706 does not interact with these hairpins, even when both the G-rich hairpin and Znf706 are present at very high concentrations (Figs. 1c and S5a-c). Next, to directly probe if Znf706 has a preference to bind a G-quadruplex structure over a duplex containing the exact same sequence and its complementary strand, a competitive NMR titration experiment was performed using a 15-nucleotide long sequence (Table 1) derived from Bcl2 promotor (Bcl2SH). Imino proton chemical shift analysis showed that for a sequence capable of forming both a G-quadruplex, as a single strand oligo, or a stable duplex, in the presence of its complementary strand, that Znf706 binds exclusively to G-quadruplexes and fails to interact with duplex DNA, even at high micromolar concentrations. This indicate that Znf706 recognizes G-quadruplex structures and not solely the G-rich nature of the sequence (Figs. 1d and S5d, e).

### G-quadruplex topology and folding influence Znf706 binding affinity

**Fig. 2 ∣:**
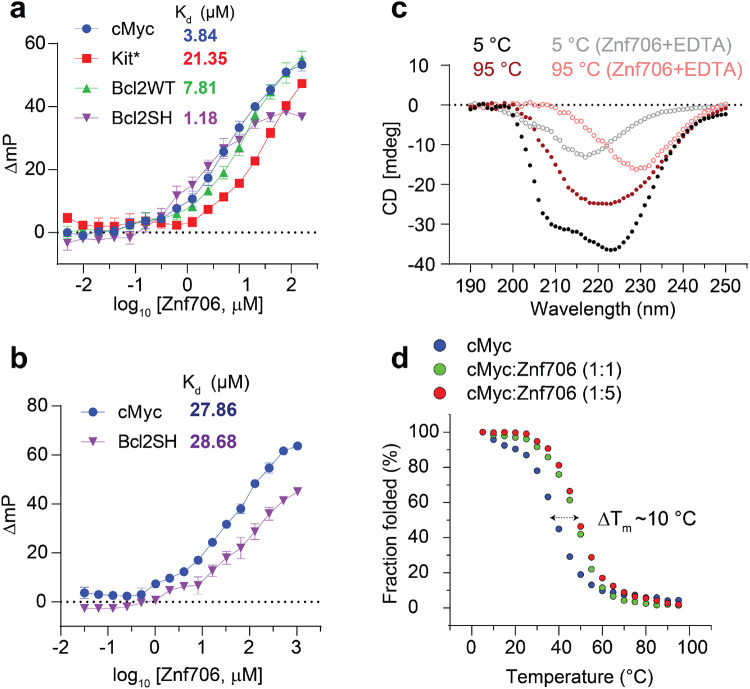
Znf706 displays thermal stability and binds more tightly to well folded G-quadruplexes. **(a-b)** FP binding assay with Znf706 and FAM-labeled G-quadruplexes prepared in 20 mM NaPi, 100 mM KCl, pH 7.4 **(a)** or 20 mM Tris-HCl, 100 mM LiCl, pH 7.4. **(b)** The indicated K_d_ values are calculated by non-linear regression analysis and one site binding saturation model in GraphPad Prism at an increasing concentration of Znf706 (5 nM to 10 μM). Error bars represent standard deviations derived from three replicates. **(c)** Secondary structure analysis of Znf706 (50 μM) in the absence (solid circles) or presence of 20x molar excess of EDTA (open circles) studied using CD spectroscopy recorded at different temperature as indicated. **(d)** Circular dichroism (CD) melting curves of 20 μM cMyc G-quadruplex in the absence (blue) or presence of equimolar (green) or 5x molar excess (red) of Znf706 dissolved in 20 mM NaPi, 4 mM KCl, pH 7.4. The CD molar ellipticity at 264 nm as a function of temperature is normalized to generate the melting curves and the CD melting temperatures (T_m_) were calculated by fitting the fraction folding curves in Origin and the arrow indicates the change in T_m_.

To better understand the physiological function of Znf706, we explored the structural specificity of Znf706’s binding properties using well characterized G-quadruplexes. The tested oligonucleotides capable of forming G-quadruplexes were derived from the promotor regions of the c-MYC (Pu19_A2A11) ^37^, BCL2, and c-KIT oncogenes, as well as the human telomeric repeat sequence (hTel) and a G4C2 repeat expansion sequence^38^ associated with amyotrophic lateral sclerosis (ALS). All of these quadruplexes have been extensively characterized (Table 1 and references therein). Using fluorescence anisotropy and microscale thermophoresis we found that Znf706 binds to these G-quadruplexes with dissociation constants (K_d_) ranging from ~ 1 to 20 μM. The strongest Znf706 binding was to Bcl2SH and cMyc, both of which form parallel type G-quadruplexes. Znf706 binds less strongly to hTel^39^ and Bcl2WT, sequences that form hybrid structures, and weaker still to Kit*, which forms antiparallel structures^40^ (Figs. 2a and S6). These observations suggest that Znf706 prefers binding parallel over hybrid G-quadruplex structures. However, since parallel G-quadruplexes have been previously shown to be more tightly folded than hybrid structures^41^ we cannot rule out the possibility that Znf706 prefers binding to tightly folded G-quadruplexes.

Consistent with the hypothesis that Znf706 interacts with G-quadruplexes in a structure-specific fashion, we observed a substantial increase in the dissociation constants (K_d_) for parallel G-quadruplex forming oligonucleotides, such as Bcl2SH and cMyc (Table 1), when they are dissolved in buffer containing LiCl, which disfavors G-quadruplex formation as compared to when they are dissolved in a KCl solution, which favors G-quadruplex formation (Fig. 2a, b). Since oligonucleotides either adopt unfolded or unstable partially folded states in the presence of LiCl, sequence specific interactions between G-quadruplexes and Znf706 are unlikely. NMR experiments further showed that a 4-repeat G4C2 G-quadruplex forming oligonucleotide has a negligible binding effect on Znf706 ^1^H/^15^N chemical shifts in the presence of NaCl as compared to in the presence of KCl, consistent with the knowledge that these oligos fold into more defined G-quadruplexes in KCl as compared to in NaCl^38^ (Fig. S7).

### Znf706 binding stabilizes and can affect the topology of G-quadruplexes

It is known that protein binding can regulate the biological activity of G-quadruplexes by stabilizing or destabilizing them or affecting their topology^42^. However, before investigating if Znf706 binding affects G-quadruplex folding we needed to first investigate how Znf706 and the G-quadruplexes fold in isolation. Thermal denaturation of Znf706 by circular dichroism (CD) showed that it is highly thermostable. It slowly unfolds and folds in a highly reversible and non-cooperative manner sometimes seen for proteins containing multiple folding intermediates^43^ (Fig. S8). This unusual heat resistance and unusual folding behavior has previously been observed for the Znf706 homologue SERF2, which has led to it being classified as a HERO, a “Heat Resistant protein of Obscure function” ^44^. Though Znf706’s temperature dependent CD spectrum indicates loss of helicity upon shift to 95 °C, Znf706 does retain a substantial amount of its β-structural characteristics even at this strongly denaturing temperature (Figs. 2c and S8). For such a small disulfide-free protein, Znf706 is thus unexpectedly thermostable. The thermal stability of Znf706 was further confirmed by using 2D heteronuclear NMR with spectra taken at temperatures ranging from 4 °C to 65.5 °C. These spectra continued to show well dispersed, essentially unchanged amide (N-H) peaks, in the range of ~7 to 9.2 ppm that corresponds to a folded protein. Loss of structure was observed when treated with the metal ion chelator EDTA, as measured both by CD and NMR spectroscopy, is consistent with Znf706’s zinc finger being important for Znf706 folding (Figs. 2c and S9). Upon EDTA addition, the amide peaks narrowed into the 7 to 8 ppm range, indicating that metal chelation leads to the structural unfolding of its thermally stable, but metal ion dependent, zinc-finger domain (Figs. S1d and S9).

In isolation, all G-quadruplexes tested were found to unfold and refold reversibly with melting midpoints (T_m_) ranging from 37 to 53 °C in low salt conditions (Figs. 2d and S10). Fortunately, these temperatures minimally impact Znf706 stability (Figs. 2d and S9a), enabling us to determine if Znf706 impacts G4 folding, without having to be concerned that Znf706 is unfolding during the measurements. To determine if Znf706 impacts G-quadruplex stability, we performed CD spectroscopy. We showed that three of the four quadruplexes tested were stabilized by Znf706 (Figs. 2d and S10). The high thermal stability of Znf706 also encouraged us to test the influence of Znf706 on the refolding of G-quadruplexes. The G-quadruplexes were denatured by heating to 95 °C, followed by rapid cooling, both in the presence and absence of Znf706. cMyc and Bcl2SH refolded very rapidly into parallel structures and Znf706 had no effect on their refolding. However, the presence of Znf706 resulted in Bcl2WT and Kit* refolding into hybrid like structures with an increased content of antiparallel-like structures. This was evidenced by a decrease in the 260 nm and an increase in 295 nm CD peak intensities compared to the spectra that were measured in the absence of Znf706 (Fig. S11). This indicates that Znf706 is capable of not only stabilizing G-quadruplexes but also altering their folding behavior.

### cMyc G-quadruplex binding induces conformational rigidity and rearrangement of Znf706

**Fig. 3 ∣:**
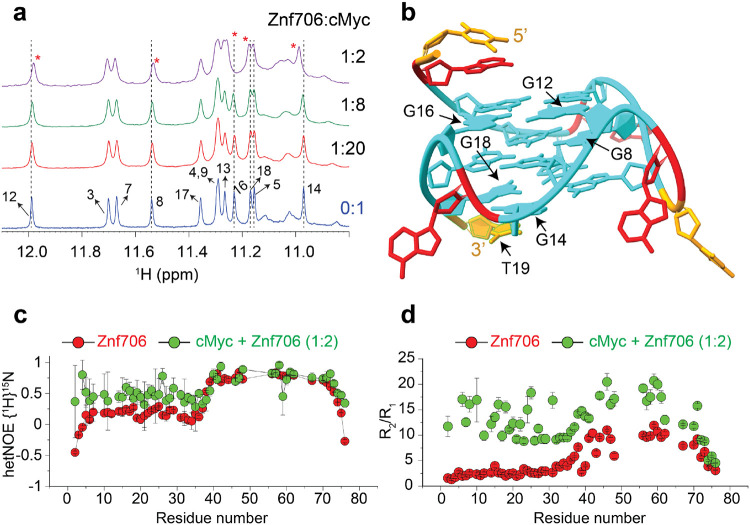
NMR guided structural and dynamic studies showing cMyc G-quadruplex binding induces conformational rigidity in the N-terminal SERF domain of Znf706. **(a)** 1D ^1^H NMR spectra showing the imino proton chemical shifts in cMyc (500 μM) in the absence (blue) and in the presence of an increasing concentration of Znf706 as indicated. The G-imino peaks that show significant chemical shift changes (G8, G12, G14, G16, and G18) are marked with dashed line and asterisks. **(b)** A high-resolution structure of cMyc (PDB ID: 2LBY) with the nucleotides likely involved in the binding with Znf706 and/or undergo conformational alteration upon Znf706 binding are marked (G8, G12, G14, G16, G18, T19). A, T and G nucleotides are shown in red, orange, and cyan, respectively. **(c)** hetNOE measurement of 200 μM ^15^N Znf706 in the absence (red) and presence (green) of 100 μM cMyc, showing the induction of structural rigidity in Znf706’s N-terminal upon cMyc binding. Standard errors are estimated from the signal-to-noise ratios. **(d)** R_2_/R_1_ relaxation ratios of 200 μM Znf706 in the absence (red) and presence (green) of 100 μM cMyc G-quadruplex. The hetNOE and relaxation NMR experiments were done on a Bruker 600 MHz at 4 °C in 20 mM NaPi, 100 mM KCl, pH 7.4 buffer containing 7.5% D_2_O.

Despite the significance role G-quadruplexes are known to have in gene regulation and in affecting protein aggregation, and the importance of protein binding in the formation and stabilization of G-quadruplexes in vivo, relatively little is known either about how proteins interact with G-quadruplexes^22,27^ or the role that protein dynamics may play in controlling protein and G-quadruplex interaction^38,45,46^. NMR titration measurements showed that when Znf706 binds to the structurally well characterized cMyc and Bcl2WT G-quadruplexes^37,47^, substantial chemical shift changes occurred in the signals of guanine imino protons that were distributed across all three stacked G-quartets in both quadruplexes (Figs. 3a, b, S12, and S13). These changes could be due to Znf706 binding to multiple interfaces or Znf706 binding induced conformational changes in cMyc and Bcl2WT. However, since CD analysis indicates that the addition of Znf706 induces very little structural change in folded cMyc and Bcl2WT G-quadruplexes (Fig. S14), this disfavors the second possibility.

Our Znf706 NMR structural data put us in a good position to also investigate the effect that G-quadruplex binding has on Znf706 protein structure and dynamics. To determine if G-quadruplex binding influences the dynamics of Znf706, we exploited the ^1^H/^15^N heteronuclear Overhauser Effect (hetNOE). This approach is often used to measure local backbone dynamics. HetNOE values close to one indicate a high degree of order and values closer to zero or below zero indicates a high degree of disorder^48^. Using these criteria, the N-terminal residues of Znf706 were identified to be disordered, and the zinc-finger region was ordered (Table 2).

We next studied the effect parallel cMyc G-quadruplexes had on Znf706’s structure and dynamics. cMyc G-quadruplex binding was shown to substantially restrict the backbone motion of the N-terminal domain, while leaving the zinc finger’s protein dynamics minimally perturbed (Fig. 3c and Table 2). The change in disorder in Znf706 upon cMyc G-quadruplex binding was further investigated by measuring the R_2_/R_1_ ratio of the spin-lattice (R_1_) and spin-spin (R_2_) relaxation rates (Fig. 3d). This ratio measures motion of individual residues, with lower values indicative of a fast motion and local flexibility, and higher numbers corresponding to slow motion and local rigidity. The N-terminal SERF homologous domain (residues 1-38) and C-terminal zinc finger domain (residues 39-76) of Znf706, in the absence of cMyc G-quadruplex, showed an average R_2_/R_1_ ratio of ~2.9 and ~7.8 respectively, confirming their respective fast and slow dynamics (Table 2). The motion in the N-terminal domain of Znf706 is greatly constrained upon cMyc binding as the average R_2_/R_1_ value for this region increases from ~2.9 to ~12.3 and the zinc-finger domain is also somewhat constrained (an increase in the R_2_/R_1_ ratio from ~7.8 to ~13.9) indicating that both the N- and C-terminal domains of Znf706 may coordinate with one another in binding cMyc G-quadruplexes (Fig. 3d).

### Mapping G-quadruplex binding sites in Znf706

**Fig. 4 ∣:**
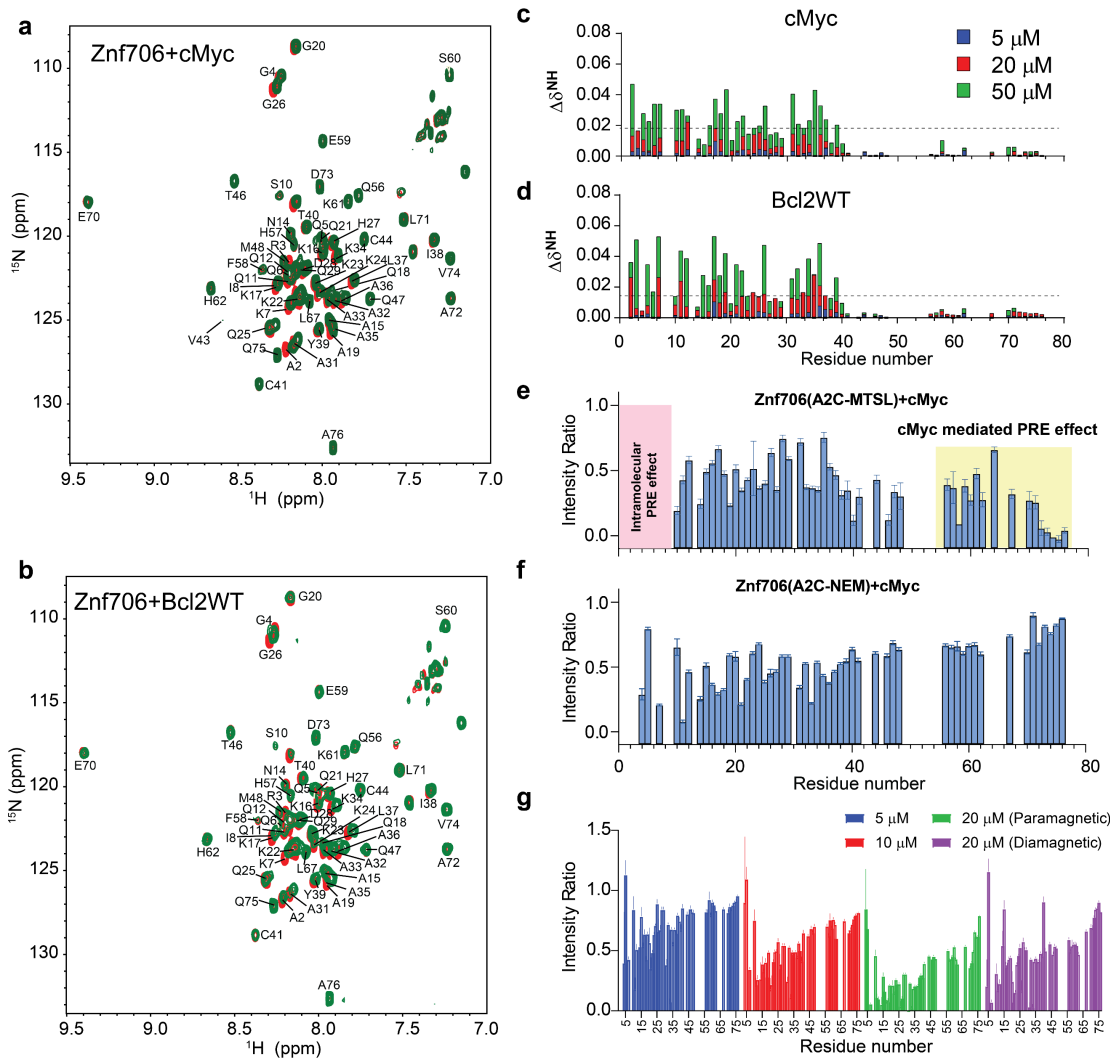
Heteronuclear and PRE NMR studies showing that Znf706’s N-terminal SERF predominantly binds to G-quadruplex and its C-terminal zinc-finger domain facilitates complex formation. **(a-b)** ^15^N/^1^H 2D correlation spectrum of 100 μM uniformly ^15^N labelled Znf706 dissolved in 20 mM NaPi, 100 mM KCl, pH 7.4 in the absence (red) and presence of equimolar (green) cMyc **(a)** and Bcl2WT G-quadruplexes **(b**). The non-proline backbone amide resonances are assigned using a series of 3D NMR experiments that include HNCA, HNCACB, CBCACONH, HNCO, and HNCOCA and 2D ^15^N- and ^13^C-HSQC. **(c-d)** Plots showing chemical shift perturbations (CSPs) derived from the ^15^N/^1^H 2D spectrum of 100 μM Znf706 titrated with variable concentrations of cMyc **(c)** and Bcl2WT **(d)** G-quadruplexes as indicated in colors. The CSPs are calculated using equation $\Delta \delta_{NH}=\sqrt{{\left(\delta^{1}H\right)}^{2}+0.154\times {\left(\delta^{15}N\right)}^{2}}$ and the dashed lines indicate the average CSPs in each group. **(e-g)** Signal intensity ratio of amide protons observed for 100 μM ^15^N Znf706 A2C-MTSL **(e),** Znf706 A2C-NEM **(f)** mixed with 50 μM cMyc G-quadruplex. The intramolecular PRE effects at N-terminus are shaded in pink, whereas cMyc binding induced PRE effects are highlighted in yellow. **(g)** Monitoring the paramagnetic effect upon cMyc binding to 50 μM ^15^N Znf706 A2C-MTSL at the indicated concentration. The final titrated product containing 50 μM Znf706 and 20 μM cMyc was reduced for ~3 hours following acquisition of the diamagnetic spectrum. NMR spectra were collected either on Bruker 800 or 600 MHz spectrometer at 4 °C for samples dissolved in 20 mM NaPi, 100 mM KCl, pH 7.4 buffer containing 7.5% D_2_O. Standard errors are estimated from the signal-to-noise ratios.

We next decided to investigate the G-quadruplex binding sites on Znf706. ^1^H/^15^N 2D correlational NMR titration experiments allowed us to determine the specific residues of Znf706 that are involved in G-quadruplex binding. Addition of even substantially substoichiometric quantities of G-quadruplexes showed that chemical shift perturbations occurred predominantly in the N-terminal, SERF-homologous, regions of Znf706 (Fig. 4a, b), with the residues A2, R3, K17, L37, and V43 being most sensitive to G-quadruplex addition. That these residues were uniformity perturbed independent of the G-quadruplexes added (Figs. 4c-d and S15a), strongly suggest that Znf706 binds similarly to G-quadruplex structures independent of their sequence or topology. Increasing the amount of G-quadruplex added, results in all the N-terminal residues having significant chemical shift perturbations, additionally emphasizing the importance of this SERF homologous domain in G-quadruplex interaction (Fig. 4c, d). Upon further increases in G-quadruplex concentration to 50 μM resulted in weak but noticeable chemical shift perturbations in the very C-terminal residues (70-75) of Znf706 (Figs. 4c, d, and S15a), indicating either that both the N- and C-termini of Znf706 contribute to G-quadruplex binding, or that a conformational change in Znf706 occurs upon G-quadruplex binding. Fluorescence polarization binding analysis of individual domains showed that a peptide consisting of just the 35 N-terminal domain residues (1-35), bound with K_d_ values ranging from ~7 – 42 μM to the three tested G-quadruplexes (cMyc, Bcl2SH and Bcl2WT), which is substantially weaker than their binding to full-length Znf706. The C-terminal zinc-finger domain, on its own, showed very weak binding to all three G-quadruplexes, with K_d_’s too high to be accurately determined (> 200 μM) (Fig. S15b, c). Although the PWM predictor program indicates that Znf706’s C-terminal zinc finger interacts with polyG sequences, it on its own does not appear to be sufficient for a high affinity interaction with G-quadruplexes.

To better understand the role of C-terminal zinc-finger domain in substrate recognition, we performed NMR paramagnetic relaxation enhancement (PRE) experiments (Fig. S16). The high sensitivity of PRE NMR has often been used to measure intramolecular as well intermolecular interactions^49^. If G-quadruplex binding is solely driven by the N-terminal SERF-homologous domain, incorporation of a PRE tag, MTSL, into the N-terminus via an introduced cystine mutant, A2C should have no PRE effect on the C-terminus zinc-finger domain and vice versa. However, the addition of cMyc G-quadruplex to the PRE tagged A2C-MTSL Znf706 at 2:1 Znf706^A2C-MTSL^:cMyc ratio induced a strong PRE effect, not just locally, but also in several regions in both Znf706’s N- and C-terminal residues (Fig. 4e, yellow shading). To rule out the possibility that A2C mutation and PRE tagging might have an impact on the binding mode of Znf706 to cMyc G-quadruplex, we tested the binding effect of cMyc G-quadruplex on the same protein construct (Znf706 A2C) tagged with a non-PRE tag N-ethylmaleimide (NEM). This protein, measured under the same experimental conditions and at the same molar ratios i.e. 2:1 Znf706^A2C-NEM^:cMyc, showed very little change in the signal intensities of the C-terminal residues (70-75), in contrast to the PRE tagged version, which did have large signal intensity changes in these residues (Fig 4f). This result argues against the possibility that cMyc binding directly influences the C-terminal residues signal intensities. To further investigate this possibility in the context of Znf706^A2C-NEM^ binding to cMyc, we compared the relative change in signal intensities of corresponding residues in diamagnetic (reduced) and paramagnetic (oxidized) environments which correlates to the difference in PRE effect in Znf706 and Znf706-cMyc complex systems (Figs. 4g and 17a). First, a slow titration of cMyc to Znf706 A2C-MTSL was carried out to monitor the PRE effect on C-terminal residues 70-75. At a very low cMyc concentration i.e., 1:10 cMyc: Znf706^A2C-MTSL^ molar ratio, a substantial loss of signal intensity was observed only for the N-terminal residues. Upon increasing cMyc concentration (1:5 cMyc:Znf706), signal loss in C-terminal residues (~70-76) appeared which subsequently increased at a 1:2.5 cMyc:Znf706 ratio (Fig. 4g). Introduction of a diamagnetic environment recovered an average ~35 % loss of signal intensities for residues 70-75 implying that C-terminal residues of Znf706^A2C-MTSL^ might possibly be involved in cMyc complex formation (Figs. 4g and S17a). However, since cMyc binding to Znf706 ^A2C-NEM^ did not show a significant decrease in intensity for C-terminus residues and the diamagnetic condition did restore signal loss of the C-terminal residues (Fig. 4e-g), we concluded that the PRE induced change in signal intensities is likely not directly due to cMyc G-quadruplex binding but rather due to Znf706 structural rearrangements or oligomerization that occur upon G-quadruplex binding that result in the N- and C-termini of Znf706 being brought into close proximity. Consistent with this interpretation, a reciprocally similar PRE effect on the N-terminus residues was observed when a PRE tag was placed at the C-terminus (A76C) as in Znf706^A76C-MTSL^ (Fig. S17b).

### Znf706 and G-quadruplex interactions mediate liquid-liquid phase transitions

**Fig. 5 ∣:**
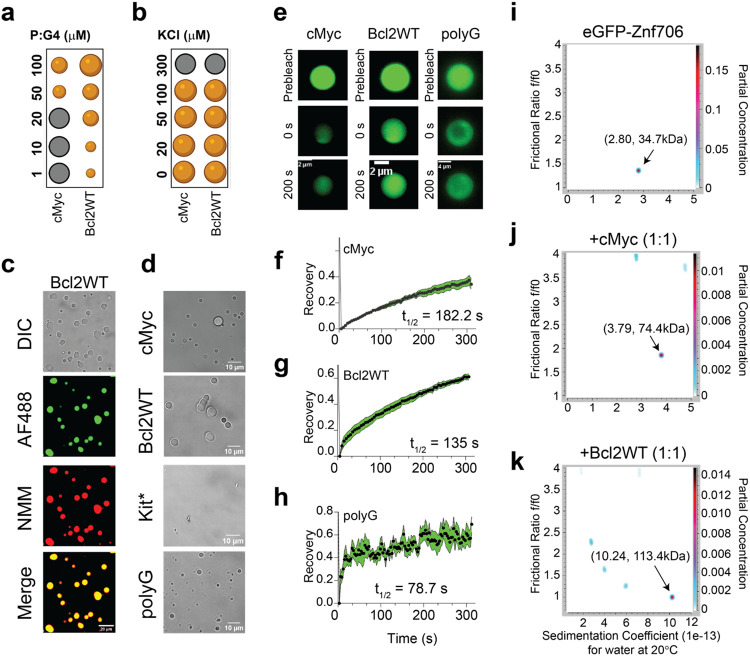
Znf706 binding to G-quadruplex forming DNA oligonucleotides induces liquid-liquid phase transition under physiological salt conditions. **(a-b)** Regime diagrams illustrating the effect of Znf706 and G-quadruplex (1:1) at the indicated concentration **(a)** and variable salt concentrations with equimolar 100 μM Znf706 and G-quadruplex **(b)** on droplet formation. Sample solutions are incubated overnight prior to imaging. **(c)** Fluorescence images of Znf706-Bcl2WT droplets prepared in 20mM NaPi, 20mM KCl, pH 7.4. The sample mixtures were prepared at room temperature by incubating 100 μM Znf706, 1 μM AF488-Znf706 (green signals), and 100 μM Bcl2WT. Droplet formation was confirmed by differential interference contrast (DIC) imaging followed by the addition of 5 μM NMM to visualize Bcl2WT G-quadruplexes (red signals). **(d)** Monitoring droplet formation of 100 μM Znf706 mixed with an equimolar amount of topologically distinct G-quadruplexes as indicated. **(e)** FRAP analysis of Znf706 droplets formed in the presence of equimolar Znf706 and G-quadruplex reveals long recovery suggesting slow Znf706 dynamics. Fluorescence images retrieved before FRAP, right after FRAP (0 sec), and at increasing diffusion times are shown. **(f-h)** Znf706- G-quadruplex droplets were half-bleached and the normalized FRAP recovery of Znf706 droplets formed in the presence of cMyc **(f),** Bcl2WT **(g),** and polyG **(h)** G-quadruplexes were fitted to a one-phase association curve to obtain the recovery half times (t^1/2^) which are indicated on the figures. Errors represent standard deviations of measurements derived from 4 droplets in each sample. **(i-k)** Determination of Znf706 and G-quadruplex complex size using analytical ultracentrifugation (AUC). eGFP-Znf706 (12.2 μM, predicted molecular weight 35.5 kDa) dissolved in 20mM NaPi, 100 mM KCl, pH 7.4 **(i)** was incubated with equimolar cMyc **(j)** or Bcl2WT **(k)** for 1 hour prior to AUC measurement (absorbance 488 nm). AUC data are analyzed in Ultrascan and a two-dimensional plot of frictional ratio f/f0 versus sedimentation coefficient S are shown with an indicated estimated molecular weight as shown by arrows. The partial concentration represented by color intensity in the z-dimension represents the abundance of each species.

Many nucleic acid binding proteins, in the presence of RNA or DNA, readily undergo liquid-liquid phase transition (LLPT) in vitro and nucleic acids are important components of cellular LLPT^50^. Proteins like Znf706 that contain low complexity regions are overrepresented in LLPT compartments and G-quadruplexes have recently been reported to accumulate in stress granules^20,51^, a LLPT compartment^52^. We were thus not surprised when we observed that upon mixing of Znf706 with G-quadruplexes that large LLPT droplets form, that upon testing were found to contain both the protein and the DNA (Figs. 5a-d, S18-21, Videos SV1 and SV2). Droplet formation was abolished at high salt concentrations (300 mM) demonstrating the importance of electrostatic interactions in driving LLPT (Fig. 5b). Droplets were formed in the presence of cMyc, Bcl2WT and polyG G-quadruplex forming oligonucleotides, but no droplets were observed in the presence of the Kit* G-quadruplex, which binds Znf706 weakly, or polyA, polyT, polyC and polyN, which do not form G-quadruplexes and bind to Znf706 very weakly or not at all (Figs. 5d and S22). To investigate the liquid-like properties of these droplets, we next carried out fluorescence recovery after photobleaching (FRAP) experiments. FRAP experiments showed that Znf706 can diffuse within the Znf706 and G-quadruplex droplets after complexing with G-quadruplex DNA, but it does so relatively slowly with half-lives varying from 80-180 s (Fig. 5e-h). Similar long FRAP recovery times are observed for proteins binding to anionic biomolecules such as nucleic acids and polyphosphates^53,54^.

### Znf706 and G-quadruplex complexes are polymorphic

Multivalent interactions are recognized as being important for biomolecular condensate formation^55-57^. Though Znf706 is a relatively small protein, our 2D correlational NMR observations suggest that its SERF-homologous and zinc-finger domains might be directly and indirectly involved in G-quadruplex recognition as evidenced from a strong and weak chemical shift perturbations, respectively (Fig. 4c). N-terminal residues 2-37 are predominantly involved in the G-quadruplex binding, whereas the C-terminal residues (70-75) are spatially rearranged, likely stimulating complex formation as evidenced from the PRE analysis (Fig. 4e).That both domains accompanied by a substantial PRE effect experienced by residues located in one terminus in the presences of a PRE tag on the other terminus, suggesting that G-quadruplex binding may mediate either structural rearrangement of Znf706 or its oligomerization (Fig. 4f, g). In isolation, G-quadruplexes showed multiple signals as tested using analytical ultracentrifugation (AUC) and size-exclusion chromatography, indicating the presence of multiple different oligomer structures (Figs. S23 and S24). However, these oligomers resolved into a single higher molecular weight species in the presence of Znf706, a species that contains Znf706 (Figs. 5i-k and S23). Size calculations show that Znf706 in the presence of an equimolar amount of cMyc G-quadruplex, results in the formation of a 2:1 protein:cMyc complex. In the presence of the Bcl2WT G-quadruplex, however, larger more polymorphic complexes were detected (Fig. 5i-k) indicating that in this case, that G-quadruplex binding induces oligomerization.

### Znf706 colocalizes with G-quadruplexes in vivo

**Fig. 6 ∣:**
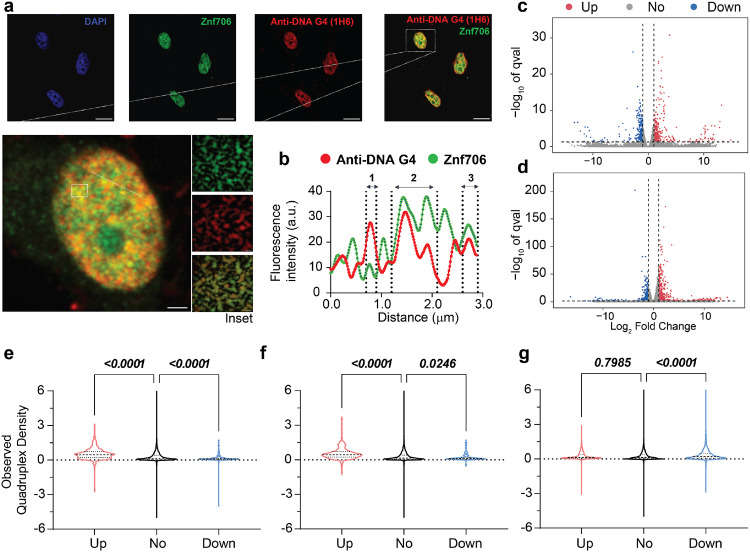
Znf706 co-localizes with DNA G-quadruplexes and its depletion alters gene expression in vivo. **(a)** Formal-dehyde fixed HEK293T cells were immunolabeled and counterstained with DAPI, anti-Znf706, and anti-DNA G-quadruplex binding specific antibodies, clone 1H6, at the indicated colors. A zoomed single cell with an inset highlighting the Znf706 and DNA G-quadruplex colocalization (yellow signals) is shown. **(b)** The degree of Znf706 and G-quadruplex localization was calculated by quantifying the red and green fluorescence signal intensities across a straight line drawn along a selected cell as shown in zoom using ImageJ. Scale bar represents 10 μm. (**c-d**) Volcano plots of RNA-seq differential expression analysis in Znf706 knockdown HeLa (c) and HEK293T (d) cells compared to control knockdown. The genes with a minimal fold change in gene expression levels are represented within the dashed vertical lines. Znf706 depletion down- and up-regulates 458 and 696 genes in HeLa, and 294 and 405 genes in HEK293T cells, respectively, and are indicated on left and right side in the volcano plots. **(e-g)** Violin plots of observed G-quadruplex density of annotated genes from Znf706 knockdown RNA-seq in HeLa (e) and in HEK293T (f) cells. A similar analysis was done to generate a violin plot for the relationship between G-quadruplex density and genes affected by a DHX36 knockout in HEK293 cells using the data obtained from work done by Chambers et al^17^.

To investigate if Znf706 and G-quadruplex interactions occur in vivo, we studied the colocalization of endogenously expressed Znf706 and DNA G-quadruplexes in human embryonic kidney (HEK) 293T cells. DNA G-quadruplexes are abundantly distributed in the nucleus of cells such as HeLa, U2OS, K562, HaCaT, hESCs, MEFs, and H1299 as visualized using different approaches^19,51,58-61^. Znf706 stained in green using an anti-Znf706 antibody (Fig. S25a) was almost exclusively localized to the nucleus, as were DNA G-quadruplexes stained in red using the anti-DNA G-quadruplex antibody (Fig. 6a). We further confirmed the colocalization using the intensity profiles obtained from the 2D single-cell image analysis (Fig. 6b) which showed a significant signal intensity overlap between Znf706 (green) and anti-DNA G-quadruplex antibody, clone 1H6 (red). The Pearson’s correlation coefficient between DNA G-quadruplexes and Znf706 staining was 0.43± 0.13, very similar to the correlation coefficients previously observed between anti-DNA G-quadruplex staining and known DNA G-quadruplex binding proteins such as TRF1, acetylated H3K9, demethylated H3K9, PML bodies and RNA polymerase II which have in vivo Pearson’s correlation coefficients with G-quadruplex antibody ranging from 0.15 to 0.68 (Figs. 6b and S25b)^58^. We conclude that Znf706 likely colocalizes with G-quadruplexes in vivo.

### G-quadruplexes suppress the effects of Znf706 on protein aggregation

**Fig. 7 ∣:**
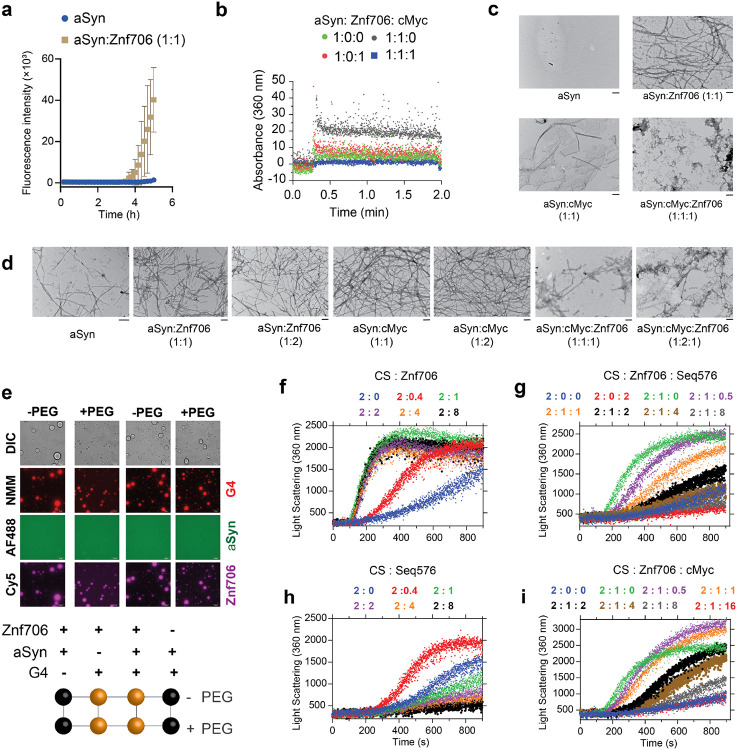
G-quadruplex binding suppress the aggregase activity of Znf706. **(a)** Monitoring the aggregation kinetics of 100 μM α-Synuclein (aSyn) dissolved in 20 mM NaPi, 100 mM KCl, pH 7.4 mixed without (blue) or with 100 μM Znf706 (gold) by ThT fluorescence. Aggregation of aSyn in sample containing equimolar Znf706 and cMyc G-quadruplexes are not shown and carried out in the absence of ThT. **(b)** Light scattering of aSyn aggregates (10 μL of aSyn aggregates are dissolved in 1590 μL buffer) taken from (d) at time ~ 6 hours at the indicated Znf706 and cMyc concentrations. **(c)** TEM images of aSyn aggregates (10 μL) taken from **(a)** at time ~ 6 hours mixed with or without Znf706 and cMyc G-quadruplex as indicated. **(d)** TEM images of 50 μM aSyn aggregates taken at time ~48 hours (see methods) mixed with the indicated concentration of Znf706 and cMyc G-quadruplexes. The scale bar is 200 nm. **(e)** Evaluation of the effect of aSyn (100 μM) on Znf706 and G-quadruplex LLPS in 20 mM NaPi, 100 mM KCl, pH 7.4 (−PEG) and 20 mM NaPi, 150 mM KCl, 10% PEG8000, pH 7.4 (+PEG). For fluorescent imaging, 0.5 μM of AF488 labelled aSyn and Cy5 labelled Znf706 are mixed with 100 μM of unlabeled proteins. Droplet formation was confirmed by DIC imaging followed by the addition of 5 μM NMM to visualize G-quadruplexes (red signals). A phase diagram for aSyn, Znf706 and G-quadruplex mixtures in ±PEG8000 is shown in the lower panel (see images in Supporting figure 27). **(f-g)** Thermal aggregation of 300 nM citrate synthase (CS) monitored using light-scattering mixed without (blue) or with an increasing concentration of Znf706 **(f**) or Seq576 G-quadruplex **(g)** as indicated. **(h-i)** A competitive thermal aggregation assay presenting the effect of an increasing concentration of Seq576 **(h)** and cMyc **(i)** G-quadruplexes on 300 nM CS mixed with 150 nM of Znf706. The thermal aggregation experiments are performed at 48 °C in 40 mM HEPES-KOH, pH 7.5.

SERF proteins were originally isolated by their ability to accelerate aggregation of amyloid prone proteins such as α-synuclein, whose amyloid formation has been neuropathologically linked to Parkinson's disease^62^. Interestingly, G-quadruplexes have recently been reported to possess potent anti-aggregation activity^63^ and remodeling of G-quadruplex structures by α-synuclein has been demonstrated in vitro^64^. These observations led us to investigate if the previously reported pro-amyloid formation of SERF might be in some way be related to our demonstration that the SERF homologue Znf706 can interact with G-quadruplexes. We first demonstrated that Znf706, like SERF, can accelerate α-synuclein aggregation effectively, even at substoichiometric concentrations (Figs. 7a and S26a, b). We monitored this using ThT fluorescence, a dye whose fluorescence changes upon binding to amyloid fibers^65^. Determining the effect of G-quadruplexes on α-synuclein amyloid formation using this assay, however, is complicated by the observation that ThT also fluoresces upon binding to G-quadruplexes (Fig. S4). To get around this issue, we used light scattering and electron microscopy to monitor α-synuclein amyloid formation. Addition of G-quadruplexes suppresses the ability of Znf706 to accelerate α-synuclein aggregation (Fig. 7b), presumably by competing for binding. Transmission electron microscopy (TEM) imaging revealed that 100 μM α-synuclein when mixed either with equimolar Znf706 or cMyc G-quadruplexes and incubated for ~6 hours forms elongated fibrils as compared to those that just contain α-synuclein (Fig. 7c). Abundant amorphous aggregates (and a few visible fibers) were generated when α-synuclein was mixed with an equimolar concentration of both Znf706 and cMyc G-quadruplexes (Fig. 7c). TEM images taken at a longer time-point (~ 48 hours) for physiological concentration (50 μM) of α-synuclein using additional Znf706 and G-quadruplex ratios gave similar results (Fig. 7d). We found that Znf706 mixed with Bcl2WT or cMyc G-quadruplexes readily forms droplets under physiological salt concentrations, in both crowded and non-crowded conditions (Fig. 7e). However, α-synuclein mixed with Znf706 or G-quadruplexes alone does not undergo droplet formation under the conditions tested. α-synuclein showed no effect on Znf706 and G-quadruplex phase separation, and three component fluorescence analysis revealed the liquid droplets contain Znf706 and G-quadruplexes, but do not contain α-synuclein (Figs. 7e and S27). Using fluorescence polarization, we determined the relative affinity of Znf706 for G-quadruplexes and α-synuclein measured under the same buffer conditions and showed that Znf706 has a very weak binding affinity for α-synuclein, as compared to G-quadruplexes (Figs. 2a and S26c). These results are consistent with the idea that Znf706’s physiological function is more likely to be centered on G-quadruplex binding rather than on direct interactions with amyloid prone proteins like α-synuclein.

Our observations suggest that both Znf706 and G-quadruplexes can affect not just α-synuclein amyloid formation but also amorphous aggregate formation as well. We postulate that the relative stronger affinity of Znf706 for G-quadruplexes over α-synuclein allows G-quadruplexes to suppress Znf706’s ability to facilitate amyloid formation. α-synuclein can form both amyloids and amorphous aggregates and Znf706 and G-quadruplexes can affect both processes^62,63^. To simplify analysis, and inspired by the recent observation that G-quadruplexes are very efficient anti-aggregation agents in vivo and in vitro^60,61^, we decided to further investigate the effects of these macromolecules on amorphous aggregation using the classic chaperone substrate citrate synthase ^66^. Surprisingly, Znf706 was found to effectively promote citrate synthase amorphous aggregation, hinting that its molecular function in driving protein aggregation may not be limited to amyloid prone proteins (Figs. 7f and S28). On the other hand, the G-quadruplexes that include Seq576, cMyc, and Bcl2WT (Table 1) all suppress citrate synthase aggregation in the effective order of Seq576>cMyc≥Bcl2WT (Figs. 7g-i and S29). Seq576 was the most effective in anti-aggregation (Table 1) that emerged from a large screen of DNA sequences^63^, so its effectiveness as an anti-aggregation agent in our hands is expected. We next wondered if the chaperone function of G-quadruplexes is altered upon Znf706 binding. We found that the Seq576 G-quadruplexes reduce the citrate synthase aggregation that is promoted by Znf706 when present at super-stoichiometric concentrations (Fig. 7h). As compared to Seq576, the cMyc and Bcl2WT G-quadruplexes showed a relatively weak activity in suppressing Znf706’s ability to facilitate citrate synthase aggregation (Figs. 7i and S29). We conclude that Znf706 can promote both amyloid formation and amorphous aggregation and that G-quadruplexes can work in opposition to suppress both types of aggregation processes.

Given the ability of Znf706 to influence the folding of G-quadruplexes and the proposed role of these non-canonical structures in regulating gene expression, we wondered if knockdowns of Znf706 had any effect on gene expression in vivo and if this was in any way related to the presence or absence of G-quadruplexes within genes. Using RNA-seq, we found that a knockdown of Znf706 in two different cell cultures results in the significant up-regulation and down regulation of hundreds of genes. 696 annotated genes were > log2 upregulated and 458 genes downregulated in HeLa cells (Fig. 6c), while in HEK293T cells, an overlapping set of 405 genes were > log2 upregulated and 294 genes were downregulated, respectively (Fig. 6d). Interestingly, these differentially expressed annotated genes correlate positively with previously observed quadruplex density^17^. Those genes upregulated in Znf706 knockdown contain a significantly higher quadruplex density in their mRNAs, as compared to genes whose expression was not significantly changed. In contrast, genes downregulated in Znf706 knockdowns contain significantly fewer G-quadruplexes in their mRNAs or promoter regions than either the upregulated genes or the unchanged genes (Fig. 6e,f). In contrast, depletion of DHX36, which is a known G-quadruplex helicase, shows a very different correlation between gene expression and quadruplex density (Fig. 6g). In DHX36 knockout HEK293 cells, downregulated genes possess a significantly higher quadruplex density^17^. DHX36 is known to unwind G-quadruplexes in vitro^22^, and may affect gene expression effects through regulation of G-quadruplex formation in 3′ UTRs and mRNA stabilization. We have shown that Znf706, by binding G-quadruplexes, can mediate G-quadruplex stability or folding in vitro. Znf706 may be mediating the observed changes in gene expression in vivo by G-quadruplex binding events that result in changes at the transcriptional level or changes in mRNA stability.

## Discussion

G-quadruplexes appear to play important roles in regulating biological processes^67^ but how proteins bind these noncanonical structures and the impact this binding may have on G-quadruplex structure and function remains under investigation^68^. Uncovering the roles of G-quadruplexes is difficult because of the transient and dynamic nature of their formation in vivo^69^. They have the ability to switch between quadruplex structures, non-quadruplex structures, and different G-quadruplex topologies (e.g., parallel, antiparallel and hybrid). Protein binding significantly influences G-quadruplex folding and structure and thus impacts biological function^64,68^.

Here we show that Znf706 specifically recognizes G-quadruplex structures, and that binding affects the structure and dynamics of both binding partners. The human Znf706 protein recognizes different structural motifs inside of G-quadruplexes including groove regions and planar surfaces, known as G-tetrads, which are exposed on the top and bottom of a typical G-quadruplex structure. Znf706 preferentially binds G-quadruplexes with parallel or hybrid topologies, though during G-quadruplex refolding, Znf706 binding can alter G-quadruplex folding resulting in different G-quadruplex topologies. Recently, several other proteins have been identified that bind to G-quadruplexes and in doing so help to regulate their folding, stability, and biological function. For example, nucleolin^45^, FUS^38^, helicases^23,70^, and G3BP1^71^ are known to bind to G-quadruplexes and regulate their functions by stabilizing or destabilizing the G-quadruplex structures, driving phase transition^72,73^, altering their cellular localization, or by inducing stress granule formation.

Our high-resolution NMR results show involvement of the Znf706’s dynamic N-terminal domain in G-quadruplex binding (Fig. 4) in a topology-dependent manner. This highlights the importance of this evolutionary conserved domain in regulating the biological function of G-quadruplexes. Znf706 residues involved in G-quadruplex interaction map mainly to the low-complexity, highly disordered, N-terminal domain of Znf706 and G-quadruplex binding enhances order in this normally disordered N-terminal region. This adds to growing evidence that G-quadruplexes can bind disordered protein domains^74,75^. Znf706 undergoes major structural rearrangements upon binding and multiple molecules can bind to a single G-quadruplex (Figs. 4e,g and 5j,k). By displaying a limited and distinct number of binding sites, possessing only a moderate degree of disorder, and being biophysically well behaved, Znf706 and the model G-quadruplexes that it interacts with appear to present a good opportunity to understand liquid-liquid phase transition in biophysical detail. This has advantages over the study of larger, more disordered proteins which interact with each other, and their nucleic acid partners, in a less ordered and more multivalent fashion. Znf706 binding to G-quadruplexes was shown to drive LLPT (Fig. 5), a biological process increasingly being recognized as important for membranelles intracellular organization and gene expression^76,77^. Other recent studies have also linked protein binding to G-quadruplexes with phase-transition^38,78^, for example, G-quadruplexes can trigger phase transition^72,79-81^. Liquid-liquid phase transitions are known to nucleate and modulate the rate kinetics of α-synuclein aggregation^62,82^, a process that initially involves liquid droplet formation of misfolded proteins followed by their maturation into more amyloid-like gels^82,83^. We found that Znf706 and G-quadruplex form liquid-liquid phase transitions that are dynamic, undergo fusion, possess fluid-like reversible behavior, and are formed using multivalent interactions (Fig. 5). Biophysical approaches including analytical ultracentrifugation, and NMR techniques such as homo- and hetero-nuclear multidimensional experiments, paramagnetic relaxation enhancement (PRE), and relaxation NMR studies proved amenable to studying the structures and dynamics of the Znf706 and G-quadruplex complexes. This opens up the rare possibility of obtaining detailed insights into how protein and nucleic acid interactions affect both binding partners and thereby modulate LLPT. A plethora of evidence highlights the role of LLPT in exacerbating neuropathophysiology^84^ and G-quadruplexes have been linked to many folding diseases^29^. G-quadruplex forming sequences are enriched in Alzheimer’s aggregates^85^ and a number of aggregation prone proteins underlying ALS including hnRNP family proteins^86^, FUS^87^, TDP-43^88^, Ewing’s sarcoma protein^89^ , and TIA1, are all G-quadruplex binding proteins^29^. Additionally, the FMRP protein, whose loss or mutation is linked to another folding disease, Fragile X syndrome, also binds G-quadruplex structures^90^.

Znf706’s N-terminus is homologous to SERF, a protein that was originally discovered due to its ability to accelerate amyloidogenesis in disease models^11^. Given its homology to SERF, it is not surprising that Znf706 also accelerates α-synuclein fibrillation. Somewhat more surprising is our finding that Znf706 also accelerates citrate synthase amorphous aggregation (Fig. 7). This observation suggests that the SERF family of proteins may have a broader role in regulating protein folding than previously appreciated^8,12^. Our observation that Znf706 binds specifically to G-quadruplex forming sequences in combination with the recent observation that G-quadruplexes serve as potent molecular chaperones ^63^, led us to investigate the relationship between Znf706’s ability to accelerate protein aggregation and G-quadruplexes ability to inhibit that aggregation. We find that G-quadruplexes suppress Znf706’s ability to promote both α-Synuclein fibril maturation and citrate synthase amorphous aggregate formation (Fig. 7), and Znf706 suppresses G-quadruplex chaperone activity. We thus can propose a model where Znf706 and G-quadruplexes affect protein folding but in opposite ways. Znf706 knockdowns affect mRNA levels in a way that is related to the presence or absence of G-quadruplexes in the affected genes (Fig. 6). That Znf706 can affect G-quadruplex folding and stability in vitro raises the possibility that this could be the mechanism by which it regulates mRNA levels in vivo. Whether this is due to G-quadruplexes acting on transcription or mRNA stability remains to be shown. Our observations suggest that the SERF family of proteins, in conjunction with G-quadruplexes, may have a broader role in regulating protein folding and gene expression than previously appreciated.

## Supplementary Material

Supplement 1

## Figures and Tables

**Table 1.** Nucleotide sequences of G-quadruplex and hairpin DNA oligonucleotides used in this study.

Sequence name	Sequence (5’ -> 3’)	Structure
^¶^Myc (Pu19_A2A11)	TAG GGA GGG TAG GGA GGG T	G4, parallel^37^
^¶^Bcl2WT	GGG CGC GGG AGG AAG GGG GCG GG	G4, hybrid^47^
^¶^Bcl2SH	AGG AGG GGG GCG GGA	G4, parallel
Bcl2SH duplex	5’-AGG AGG GGG GCG GGA-3’	duplex
	3’-TCC TCC CCC CGC CCT-5’	
^¶^Kit*	GGC GAG GAG GGG CGT GGC CGG C	G4, hybrid^40^
^¶^G4C2 repeat	GGG GCC GGG GCC GGG GCC GGG GCC	G4, parallel^38^
C4G2 repeat	CCC CGG CCC CGG CCC CGG CCC CGG	n.d.

**Table 2.** Summary of relaxation rates and hetNOE for Znf706 N- and C-terminal domains.

Znf706 (residues)	R_1_ (S^−1^)	R_2_ (S^−1^)	hetNOE
Znf706 (1-38)	1.61±0.10	4.71±0.07	0.17±0.06
Znf706 (39-76)	1.51±0.10	11.83±3.91	0.67±0.17
Znf706 (1-38) + cMyc	1.35±0.07	16.70±4.25	0.46±0.11
Znf706 (39-76) + cMyc	1.28±0.10	17.82±5.49	0.68±0.19
