# Supplementary material for: Effects of protein G-quadruplex interactions on phase transitions and protein aggregation
Source: bioRxiv. 2023 Sep 21:2023.09.21.558871. Preprint. [Version 1] doi: 10.1101/2023.09.21.558871 (PMC10542165; doi:10.1101/2023.09.21.558871)
Supplement: Supplementary file 1 [file media-1.pdf]

# Supplementary Information

## Effects of protein G-quadruplex interactions on phase transitions and protein aggregation

Bikash R. Sahoo,[1,2] Vojč Kocman,[3] Nathan Clark,[1,2] Nikhil Myers,[1,2] Xiexiong Deng,[1,2] Ee L. Wong,[1,2] Harry J. Yang,[1,2] Anita Kotar,[3] Bryan B. Guzman,[4] Daniel Dominguez,[4] Janez Plavec,[3] and James C.A. Bardwell[1,2,*]

[1]*Howard Hughes Medical Institute, University of Michigan, USA,*

[2]*Department of Molecular, Cellular and Developmental Biology, University of Michigan, Ann Arbor, MI, USA*

[3]*National Institute of Chemistry, University of Ljubljana, Slovenia*

[4]*Department of Pharmacology UNC Chapel Hill, USA*

**Table of Contents**

1. Material and methods…………………………………………………………….....2

1.1 Recombinant human Znf706 protein expression and purification ……….….…..2

1.2 Fluorescent and PRE labeling in Znf706……………..………………………….2

1.3 Oligonucleotide synthesis and G-quadruplex formation…..……………………….2

1.4 Fluorescence polarization and anisotropy……………………………………….3

1.5 Microscale thermophoresis ……………………….……………………….3

1.6 CD Spectroscopy ……………………………………….…………………….3

1.7 Analytical Ultracentrifugation (AUC) …………………………………………….3-4

1.8 NMR Spectroscopy ……………………………………………….…………….4-5

1.9 Immunofluorescence and Microscopy ……………………………………..…...5-6

2.0 Western blot analysis ……………………………………………………………6

2.1 RNA interference and RNA-seq ……………………………………………………6

2.2 α-Synuclein fibrillation and protein aggregation assay ……………………..………6-7

**Figure S1-29**……………………….……………………………………..…….……8-36

**Supplementary videos SV1-SV2**……………………………………….…..…....36

**References** ……………………….…………………………………..…….……..36-37

**Materials and methods**

**1.1 Recombinant human Znf706 and a-Synuclein protein expression and purification**

The human Znf706 sequence was codon-optimized for *E. coli* expression and cloned into the pET28 derived vector containing an N-terminal $His_6$-SUMO tag (pET-HisSumo) or an enhanced green fluorescent protein tag (eGFP). Wild-type Znf706 protein or variants containing, A2C, or A76C substitutions of were expressed as $His_6$-SUMO fusion proteins. $His_6$-SUMO and eGFP tagged Znf706 proteins were expressed in *E. coli* BL21 DE3 using PEM media at 37 °C until the culture density reached $OD_{600}$ of 1.0 followed by an overnight induction with shaking (160 rpm) using 0.2 M IPTG at a culture temperature of 22 °C. For recombinant expression of uniformly labelled $^{15}N$ or $^{15}N$,$^{13}C$ $His_6$-SUMO tagged Znf706, M-9 minimal media supplemented with $^{15}N$ and $^{15}N$,$^{13}C$ isotope labelled ammonium chloride ($^{15}NH_4Cl$) and glucose (D-Glucose-6-$^{13}C$) as sole nitrogen and carbon sources, respectively, were used instead of PEM media. Isotopes were purchased from Cambridge Isotope Laboratories. Tagged proteins were purified by passage through a nickel affinity HisTrap column (Cytiva, #17524802) followed by HisSUMO tag cleavage with ULP1 protease in 40 mM Tris, 10 mM sodium phosphate, 300 mM NaCl, pH 8.0 and dialyzed overnight at 4 °C. The resulting completely tag free Znf706 proteins were then purified by removing the cleaved His containing tag by absorption onto nickel affinity resin and collecting the flow-through. All proteins are further purified by subjecting them to an anion exchange column (HiTrap SP, Cytiva, 17115201) followed by size-exclusion chromatography (HiLoad 16/60 Superdex S75; GE Healthcare, 17106801) as previously described.[1] The resulting proteins were of high purity (Fig. S1b). The protein sample stocks were prepared either in 20 mM sodium phosphate (NaPi, pH 7.4), 100 mM KCl, 0.05% $NaN_3$ or 20 mM Tris-HCl (pH 7.4), 100 mM LiCl, 0.05% $NaN_3$. For analytical ultracentrifugation (AUC), eGFP (A206K) and eGFP (A206K) fused Znf706 proteins were prepared using the pET-HisSumo construct. The expression and purification of α-Synuclein protein was as previously described.[1] Lyophilized α-Synuclein protein was dissolved in 20 mM NaPi, 100 mM KCl, 0.05% $NaN_3$, pH 7.4 for use in the amyloid aggregation assay. Protein concentrations were measured by UV absorption using a UV-3000 (Shimadzu) spectrophotometer, molar extinction coefficients were calculated using ExPASy ProtParam tool.

**1.2 Fluorescent and PRE labeling in Znf706**

A Znf706 cysteine mutant protein (A2C) purified as described above, was used for Alexa Fluor 488 ((AF488), CAS-A10254) or 1-Oxyl-2,2,5,5-tetramethyl-Δ3-pyrroline-3-methyl methanethiosulfonate ((MTSL), CAS: 81213-52-7) labeling. To perform the labeling, 200 μM of Znf706 (A2C) was dissolved in 20 mM Tris-HCl, 0.2 mM $ZnCl_2$, pH 8.0 and incubated with a 10-fold molar excess of AF488 or MTSL overnight at 37 °C with shaking at 250 rpm. The excess AF488 or MTSL was removed by passing the samples over a PD-10 desalting column (Cytiva, 17085101). The sample buffer was next changed to 20 mM NaPi, 100 mM KCl, 0.05% $NaN_3$, pH 7.4 by either running on a size-exclusion chromatography column (Superdex S75 10/300 GL, Cytiva) or by concentrating the sample 20 times using a 3kDa cut-off filter (Amicon®, UFC500324). The AF488 labeling efficiency was determined by UV absorbance following the manufacture's protocol. For comparison NMR studies, an analog of MTSL which has no PRE effect i.e., N-ethylmaleimide (NEM) labelled A2C Znf706 was also prepared following the above-described preparation procedure.

**1.3 Oligonucleotide synthesis and G-quadruplex formation**

Desalted G-rich DNA oligonucleotides, DNA polynucleotides (A/T/C/G/N), and G-quadruplex forming DNA and RNA oligonucleotides (Fig. S2 and Table 1) were purchased either from Integrative DNA Technology (IDT) or synthesized in-house as described below. 5' 6-FAM labelled oligonucleotide sequences were purchased from IDT as HPLC purified, desalted DNA or RNA oligonucleotides. These oligonucleotides were dissolved in nuclease-free water and further desalted by 15 cycles in nuclease free water using a 3 kDa cut-off filter (Amicon®, UFC500324) and the concentration were determined using the extinction coefficients obtained using IDT Oligoanalyzer tool. In house, oligonucleotides were

synthesized on a K&A Laborgeraete GbR H-8 DNA/RNA Synthesizer. Standard phosphoramidite chemistry was used. Deprotection was done with the use of aqueous ammonia at 55 °C for 24 h. Samples were purified and desalted by 15 cycles of concentration in water using a 3 kDa cut-off filter (Amicon®, UFC500324). The concentration of the oligonucleotides was determined from their predicted extinction coefficients, obtained from IDT Oligoanalyzer tools, using a Shimadzu UV-2600 spectrometer. Oligonucleotides were next folded in either 20 mM NaPi, 100 mM KCl, 0.05% $NaN_3$, pH 7.4 or 20-mM Tris-HCl, 100 mM LiCl, 0.05% $NaN_3$, pH 7.4 and subjected to a heating and cooling cycle using a thermal cycler heating to 98 °C for 5 minutes, then cooling from 75 to 4 °C at a rate of 1 °C/minute.

**1.4 Fluorescence polarization and anisotropy**

The binding of oligonucleotides to Znf706 were determined by measuring the change in fluorescence polarization or anisotropy using a Tecan Infinite M1000 Pro plate reader or a Cary Eclipse fluorophotometer, respectively at 25 °C. Anisotropy measurements were done using 200 nM of AF488 labeled Znf706 (A2C) and increasing concentrations of DNA, RNA oligonucleotides and G-quadruplexes. For fluorescence polarization measurements, 20 µL of sample containing 20 nM of 6-FAM labelled G-quadruplexes and increasing concentrations of unlabeled Znf706 were incubated for 1 hour prior to fluorescence polarization measurements using a 384 well plate (Corning #4514). The binding dissociation constant of G-quadruplex to Znf706 was determined by a non-linear regression curve fit, and one site binding-saturation model in GraphPad Prism. All the fluorescence assays were carried out in triplicate in 20 mM NaPi, 100 mM KCl, 0.05% $NaN_3$, pH 7.4 except for those with unfolded G-quadruplexes, which were done in 20 mM Tris-HCl, 100 mM LiCl, 0.05% $NaN_3$, pH7.4.

**1.5 Microscale thermophoresis**

20 nM of Kit* and the human telomeric G-quadruplexes hTel and TERRA (UAGGGUUAGGG UUAGGGUUAGGG) were preincubated with increasing concentrations of Znf706 in 20 mM NaPi, 100 mM KCl, 0.05% $NaN_3$, pH 7.4 for ~1 hour followed by microscale thermophoresis measurements performed at 25 °C using a Monolith NT.115 (NanoTemper) instrument using the blue filter, 10-20% LED and at 80% microscale thermophoresis power.

**1.6 CD spectroscopy**

Znf706 secondary structure was studied by a JASCO 1500 far-UV CD spectrometer using a 200 µL quartz cuvette with a pathlength of 1 mm. CD spectrum of Znf706 (50 µM) dissolved in 20 mM NaPi, 4 mM KCl, pH 7.4 was collected as an average of 8 scans. The effect of zinc coordination on Znf706 secondary structure was examined by measuring the CD spectrum of Znf706 (50 µM) preincubated with a 20x molar excess of EDTA for 15 minutes at 25 °C. Znf706 thermal stability was monitored by recording its far-CD absorptions at 208 and 222 nm as we slowly heated the sample from 5 °C to 95 °C. The CD data were collected in every 5 °C time interval (averaging 2 scans/temperature) with a 3-minute delay between each temperature scan. CD measurements of G-quadruplex samples at 20 µM, dissolved in 20 mM NaPi, 4 mM KCl, pH 7.4, were averaged from 3 scans. The melting temperature of 20 µM G-quadruplexes in the absence or presence of an equimolar or a 5x molar excess of Znf706 was determined using the same settings we used for the protein only samples, as described above. The G-quadruplex or G-quadruplex-protein sample mixtures were dissolved in 20 mM NaPi, 4 mM KCl, pH 7.4, and CD spectra were collected at 25 °C.

**1.7 Analytical Ultracentrifugation**

The sedimentation velocity of various Znf706 and G-quadruplex complexes was determined using analytical ultracentrifugation (AUC). The AUC measurements were done by comparing two different samples; type-1, which contained unlabeled Znf706, and type-2, which contained Znf706 labeled with eGFP (A206K). Type-1 samples were monitored by measuring the absorption of the cMyc G-quadruplex at 260 nm. In some cases, we needed to use eGFP(A206K) labelled Znf706, because observation of G-quadruplex complexes by AUC was limited at 260 nm since Znf706 has a single tyrosine and therefore

absorbs relatively weakly at 260 nm. To bypass this issue, in some cases we used eGFP-Znf706 and monitored the absorption at 488 nm (Type-2 samples) to filter out the G-quadruplex absorbance. eGFP(A206K) absorbs strongly at this wavelength but G-quadruplexes do not absorb at all, making it a convenient wavelength for following Znf706. 420 µL of cMyc G-quadruplex samples with an $OD_{260}$ 0.35 or 0.7 were prepared in the presence or absence of either an equimolar or a 2x molar excess of Znf706. All components were dissolved in 20 mM NaPi, 100 mM KCl, 0.05% $NaN_3$, pH 7.4. Samples were loaded onto epon-charcoal two-channel centerpieces in an An60Ti rotor in a Beckman Optima Xl-I analytical ultracentrifuge using a 1.2-cm path length. AUC measurements were carried out in the intensity mode at 58,000 rpm. Type-2 sample measurements were done at 32,000 rpm at 22 °C. The sedimentation velocity data were analyzed using Ultrascan III software (version 4) and exported to the LIMS server. The data fitting was done on the LIMS server using the computing clusters available at the University of Texas Health Science Center and XSEDE sites at the Texas Advanced Computing Center. The partial specific volume of Znf706 and different G-quadruplex sequences were estimated within UltraScan III. Raw AUC intensity data were converted into pseudo-absorbance. Two-dimensional sedimentation spectrum analysis was performed to subtract time-invariant noise, followed by fitting the radially invariant noise within a S range of 1 to 10, using 64 S-value grid points and 64 f/f0 grid points. The two-dimensional sedimentation spectrum analysis was iterated 10 times after fitting the meniscus. The two-dimensional sedimentation spectrum analysis data resolution was next refined by employing genetic algorithm parameters to remove false positive data using 50 Monte Carlo iterations.

**1.8 NMR spectroscopy and Znf706 structure modeling**

NMR measurements were done for Znf706 dissolved in either 20 mM NaPi, pH 7.4 or 20 Tris-HCl as specified. 1D proton, 2D $^{15}$N-1H TROSY experiments using 16 scans, 100 t1 increments, and a 1.5 s delay, or $^{1}$H/$^{13}$C HSQC experiments using 16 scans, 128 t1 increments, and a 1.5 s delay and 3D (HNCA, HNCACB, CBCACONH, HNCO, and HNCOCA) heteronuclear NMR experiments were carried out on a Bruker 800 MHz spectrometer equipped with a triple-resonance cryoprobe and operated by Topspin 4.1.4. A sample volume of 275 µL containing 0.75 mM of $^{15}$N-$^{13}$C Znf706 dissolved in 20 mM NaPi, 20 mM NaCl pH 7.4 and 7.5% $D_2O$ filled into a Shigemi tube was used for the 2D and 3D NMR data collections. All NMR spectra were recorded at 4 °C, processed in Topspin 4.1.4 and analyzed using NMRFAM-Sparky. The Znf706 backbone assignments were done in NMRFAM Sparky. 1H NMR of G-quadruplex (50 µM or 200 µM) in different buffer and salt conditions (as specified in results) in the absence or presence of variable Znf706 concentrations were acquired (1 s delay, ns). 200 µM $^{15}$N Znf706 in the absence or presence of 100 µM cMyc G-quadruplex was used for R1, R2, and hetero-NOE type relaxation studies. $^{15}$N relaxation experiments were carried out at 4° C on a Bruker 600 MHz spectrophotometer. NMR spectra used for R1 analysis were collected by using the following relaxation delay times: 20, 50, 90, 130, 200, 320, 450, 580, 750, 900, 1200, and 2005 ms. R2 data were collected using the following relaxation delays: 16.96, 50.88, 84.80, 135.68, 169.60, 220.48, 305.28, 373.12, 457.92, 542.72, 678.40, and 1356.80 ms. The relaxation data were processed using TopSpin 4.1.1. The R1 (spin-lattice) and R2 (spin-spin) relaxation rates were determined by separating the resonance heights and fitted as a function of the relaxation delay time using NMRFAM Sparky. The hetero NOE data were collected with a relaxation delay of 2.5 s and spectra were analyzed using Sparky. Paramagnetic relaxation enhancement (PRE) NMR measurements were done using 100 µM of $^{15}$N labelled A2C-MTSL Znf706 dissolved in 20 mM NaPi, 100 mM KCl, 7.5% $D_2O$, 0.05% $NaN_3$ (w/v) pH 7.4 at 4° C mixed with and without 50 µM of cMyc G-quadruplex DNA. For a comparative study, uniformly $^{15}$N labeled Znf706 A2C mutants were tagged with N-ethylmaleimide (NEM) and NMR data were recorded on samples containing 100 µM of A2C-NEM Znf706 mixed with 50 µM of cMyc G-quadruplex. NMR spectra of 100 µM Znf706-A2C-MTSL were recorded in paramagnetic conditions following incubation with 5x sodium ascorbate for 3 hours to obtain the corresponding diamagnetic spectrum. To compare the effect of cMyc binding on Znf706-A2C-MTSL's paramagnetic effects, $^{15}$N/$^{1}$H heteronuclear spectra were recorded first for 50 µM Znf706-A2C-MTSL titrated with 5, 10 and 20 µM of cMyc G-quadruplexes. Next, the last titrated sample containing 50 µM Znf706-A2C-MTSL and 20 µM cMyc G-quadruplexes

was reduced using 20 mM sodium ascorbate for ~ 3 hours and NMR spectrum was acquired in the diamagnetic condition. NMR data were collected using a triple resonance cryoprobe equipped 600 MHz or 800 MHz Bruker spectrometers as specified in results wherever needed. All NMR spectra were processed in Topspin 4.1.1 and data analysis was done using NMRFAM-SPARKY 1.47.

The 3D model structure of Znf706 was built using the backbone chemical shifts ($C_\alpha$, $C_\beta$, N, NH, and CO) derived from 3D NMR assignments by CS-ROSETTA. The CS-ROSETTA model building using Znf706 backbone chemical shifts were successful in generating a converged 10 low energy structures of the folded non-flexible zinc-finger (ZNF), but CS-ROSETTA failed to converge for a full-length structure due to the flexible N-terminal SERF (N-SERF) characterized with high dynamics. Therefore, to build a full-length structure, the lowest energy CS-ROSETTA structure of the zinc-finger domain (39-76) was appended to the lowest energy CS-ROSETTA structure of the dynamic N-terminus (1-38) using the advanced modeling tutorial in MODELLER 10.1[2] The appended structure was next superimposed with the CS-ROSETTA derived zinc-finger domain to ensure no structure change followed by comparing it with the NMR structure of the C2H2 type zinc-finger 12 Miz-1. The zinc ion was coordinated to Znf706 or the zinc-finger domain (39-76) using the HADDOCK server using a set of unambiguous restraints.[3]

**1.9 Immunofluorescence and Microscopy**

HEK 293T/17 cells (ATCC® CRL-11268™) derived from human embryonic kidney were were grown in DMEM media supplemented with 2 mM glutamine, 1x Penicillin-Streptomycin solution (Gibco™ 10378016), and 10% fetal bovine serum (FBS). Cells (~30, 000) were seeded onto coverslips and grown overnight in 24 well plates. Cells were fixed with 4% paraformaldehyde (Electron Microscopy Science) in PBS for 10 min, washed three times with PBS, permeabilized with 0.1% Triton X-100 in PBS for 30 mins, and blocked with 1% BSA/PBS solution for 1h. All steps were carried out at room temperature. Thereafter, cells were incubated with anti-Znf706 rabbit polyclonal primary antibody (1: 200 dilutions; catalog no. AV32987, Sigma-Aldrich) and the mouse anti-DNA G-quadruplex structure antibody – clone G4 (Sigma Aldrich, MABE917), which was diluted 500-fold in blocking buffer and incubated overnight at 4°C, and then washed 3 times with PBST (0.1% Tween). Cells were next incubated with the Znf706 specific secondary antibody (Goat anti-Rabbit Alexa Fluor™ 488, Thermo Fisher, Cat# A-32731) and anti-DNA G4 specific antibody (Goat anti-Mouse Alexa Fluor™ 647, Cat#A-21235) for visualization. For co-localization studies, slides were mounted with ProLong Gold Antifade reagent with DAPI (Invitrogen). Images were captured using a Leica TCS SP8 STED 3X confocal microscope with a 100x oil objective.

The formation of liquid-liquid phase transition (LLPT) droplets that could possibly occur by combining Znf706 and G-quadruplexes and additional oligonucleotides were studied. 16-well CultureWell™ ChamberSLIP cover glasses were coated with 5% Pluronic® F-127 copolymer surfactant overnight followed by 10 washes with 0.3 ml sample buffer (20 mM NaPi, 100 mM KCl, 7.5% $D_2O$, pH 7.4) and then used for imaging. Variable concentrations of Znf706 (1, 10, 20, 50, and 100 μM) prepared in 20 mM NaPi, 100 mM KCl, 7.5% $D_2O$, pH 7.4 was mixed with an equimolar concentration of G-quadruplexes to monitor the effect of protein and G-quadruplex concentrations on droplet formation. The effect of salt on Znf706 and G-quadruplex droplet formation was studied at fixed Znf706 and G-quadruplex (100 μM) concentrations and variable salt concentrations of 0, 20, 50, 100 and 300 mM KCl. Formation of droplets was also tested for 100 μM Znf706 mixed with A, T, G and C polynucleotides. All protein and G-quadruplex mixed samples were incubated overnight in 5 mm thin-walled glass NMR tubes and then transferred to 16-well trays followed by DIC or fluorescence imaging. Droplet formation of 100 μM Znf706 mixed with 50 μM of cMyc was monitored at different timepoints (1, 5 and ~18 hours) using Differential Interference Contrast (DIC) microscopy imaging. 100 μM of Znf706 labelled with A2C-MTSL or Znf706-A2C-NEM was incubated with 50 μM of cMyc for 5 hours to test LLPT formation. To monitor the effect of α-Synuclein on liquid droplet formation of Znf706 and G-quadruplexes, 100 μM Znf706, 100 μM α-Synuclein and 100 μM cMyc/Bcl2WT G-quadruplexes were incubated for ~1 hour in a non-crowding buffer 20 mM NaPi, 100 mM KCl, pH 7.4 or a crowding buffer 20 mM NaPi, 100 mM

KCl, pH 7.4, 10% PEG8000. For fluorescence imaging and FRAP analysis, 1/100$^{th}$ of AF488 Znf706 were spiked into the sample mixture and droplet formation was confirmed using DIC and green signals from the AF488 tagged protein. Next, to visualize the G-quadruplexes with the droplets, 5 µM of N-methyl mesoporphyrin IX (NMM) was added to each sample well and mixed gently, incubated for ~10 minutes following by imaging. All imaging was done on a Nikon Ti2-E motorized inverted microscope controlled by NIS Elements software with a SOLA 365 LED light source using a 100X oil immersion objective. Fluorescence images and FRAP data analysis were done using Fiji ImageJ.

**2.0 Western blot analysis**

HEK 293T were cultured in 60 mm dishes and were washed with 1x PBS and harvested using trypsin-EDTA (0.25% trypsin, 1 mM EDTA, Gibco). Pellets were then lysed with 2x SDS sample buffer and separated by 4-15% gradient SDS-PAGE. The following antibodies were used: rabbit anti-Znf706 (catalog no. AV3298, Sigma-Aldrich at 1:1000 dilution and rabbit anti-GAPDH (catalog no. G9545, Sigma Aldrich) at 1:2500 dilution. The secondary antibody used was LICOR IRDye 800 CW goat anti-rabbit IgG (H+L) Biosciences, 926-32211; 1:5000 dilution). Blotted membranes were scanned with a LI-COR scanner. Western blot quantification analysis was performed with Image Studio Lite (LI-COR).

**2.1 RNA interference and RNA-seq**

For transient siRNA knockdown, HEK 293T and HeLa cells were grown in DMEM media supplemented with 2 mM glutamine, 1x Penicillin-Streptomycin solution (Gibco™ 10378016), and 10% FBS. The cell passage numbers were kept between 5-15. 30,000 cells were seeded onto a 6-well plate and reversely transfected with 25 nM of either non-targeting control siRNA (Horizon Discovery, D-001206-13-20) or SMARTpool siRNA targeting Znf706 (Horizon Discovery, M-021025-01-0005) using Lipofectamine RNAiMAX (Invitrogen, 13778150). Transfection media was exchanged after 24 hours, and cells were then harvested after ~48 hours knockdown. Total RNAs were extracted using Trizol reagent (Invitrogen, 15596026) according to the manufacturer manual.

Total RNAs were subjected to poly(A) RNA-seq sequencing service from LC Science LLC (Houston, Texas, U.S.A.). Paired-ended 150 bp sequencing was performed on Illumina's NovaSeq 6000 sequencing system. Three biological replicates were sequenced for each condition in different cell lines. Sequencing reads were trimmed by Cutadapt[4] and quality checked using FastQC (http://www.bioinformatics.babraham.ac.uk/projects/fastqc/). Trimmed reads were then mapped to human genome hg38 using HISAT2[5]. StringTie[6] was used to assemble transcriptome for each sample and to calculate fragments per kilobase of exon per million (FPKM) of mRNAs. Differential expression analysis of mRNAs was performed by DESeq2[7]. Those mRNAs with the parameter of false discovery rate/q-value ≤ 0.05 and absolute fold change ≥ 2 were considered differentially expressed mRNAs. The observed quadruplex density of human genome is derived from the Supplementary Table 6 in Chambers et al. 2015[8]. For comparative analysis of the quadruplex density of human genome obtained in Znf706 knockdown HEK 293T cells, we retrieved the data published by Chambers et al. 2015[8] for DHX36 helicase knockout in HEK293 cells.

**2.2 α-Synuclein fibrillation and protein aggregation assay**

The α-Synuclein aggregation assay was carried out using our previously published protocol.[1] Briefly, the aggregation kinetics of α-Synuclein were monitored using a Thioflavin-T (ThT) fluorescence assay (Excitation=440 nm; Emission=485 nm) using a half-area 96-well plate (Corning #3881). 100 µM or 50 µM α-Synuclein protein dissolved in 20 mM NaPi, 100 mM KCl, pH 7.4 was mixed with 25 µM or 12.5 µM ThT, respectively, and variable concentrations of Znf706 and cMyc G-quadruplexes (25, 50 and 100 µM). ThT was added to all sample wells except those containing G-quadruplexes. Two 3-mm glass beads were added to each sample and aggregation kinetic measurements were recorded at every 600 seconds

under continuous agitation (orbital shaking, 3mm amplitude, 600 s) at 37 °C using a Tecan Infinite M200 plate reader.

Porcine citrate synthase was purchased Sigma #C3260-5KU) and prepared for thermal aggregation using a method as described elsewhere.[9] Briefly, 1 mL of citrate synthase was dialyzed in 50 mM Tris-HCl, 2 mM EDTA, pH 8.0 at 4 °C overnight, and then concentrated using a 10-kDa cut-off filter. The protein concentration was then measured using an extinction coefficient (ε280=78060 $M^{-1}$. $cm^{-1}$) and thermal aggregation was performed using 300 nM citrate synthase dissolved in 40 mM HEPES-KOH, pH 7.5 buffer at 48 °C under constant stirring in the absence or presence of a variable amount of Seq576, Bcl2WT and cMyc G-quadruplexes. The effect of a variable concentration of Znf706 (0.06 - 1.2 μM) on citrate synthase (300 nM) thermal aggregation was monitored, and final aggregate product images were taken using DIC. Light scattering was measured using a Hitachi F-4500 fluorometer with both excitation and emission wavelengths set to 360 nm and a slit width of 1 nm.

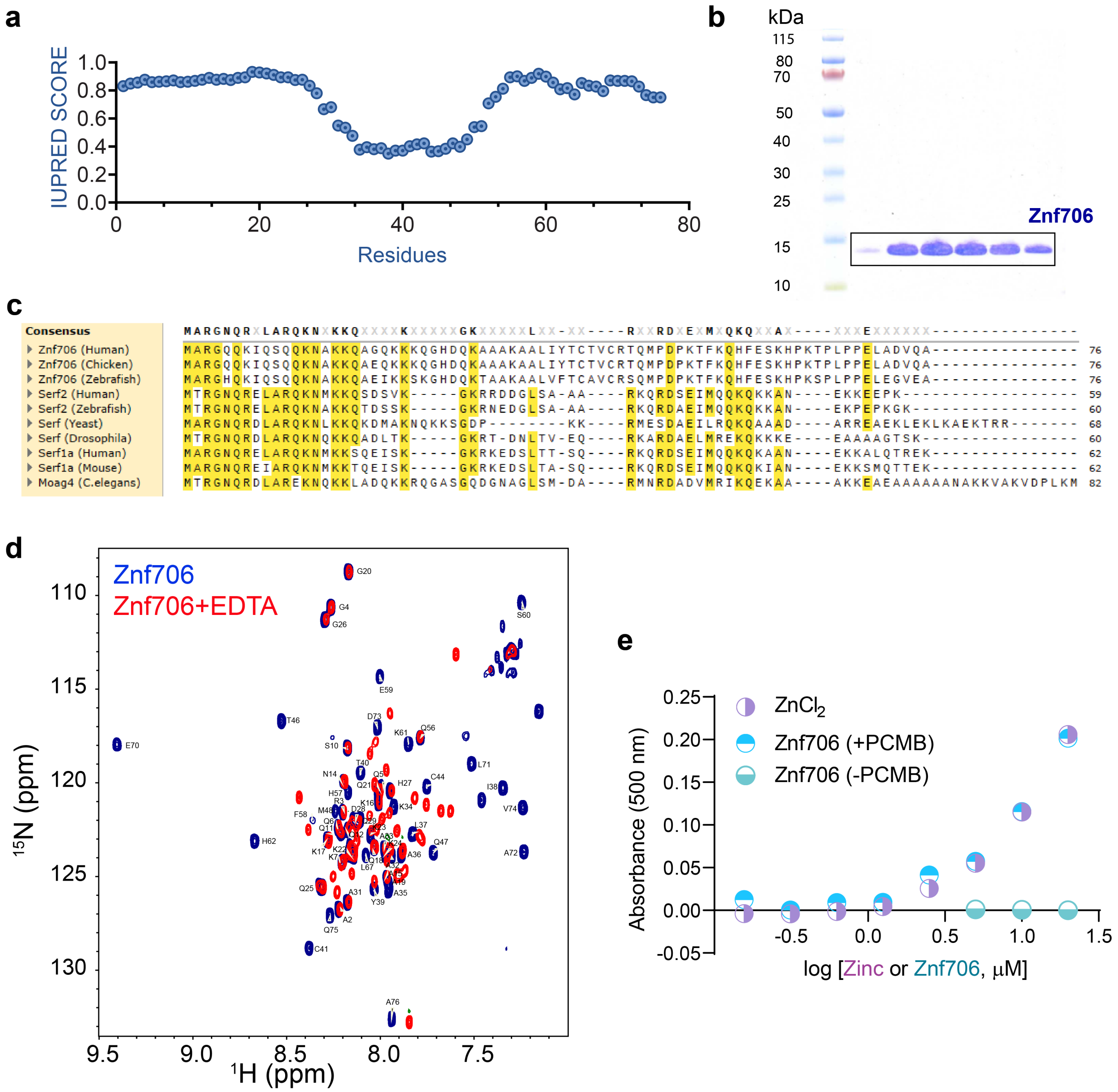


**Fig. S1**. (**a**) Prediction of the disordered regions in Znf706 using IUPred2A, with disordered regions indicated by a high IUPred score. **(b)** Recombinantly expressed and purified human Znf706 as analyzed by SDS-PAGE and Coomassie Blue staining, box indicates the pooled fractions. **(c)** Sequence alignment of human Znf706 with different SERF proteins generated using Clustal Omega. Identities are highlighted in yellow, and the conserved residues are shown on the top. **(d)** $^{15}$N/$^{1}$H TROSY experiments of 50 μM Znf706 dissolved in 20 mM NaPi, 100 mM KCl, 0.05% $NaN_3$, pH 7.4 in the absence (blue) or presence of 100x molar excess of EDTA (red) recorded at 4 °C. **(e)** Estimating the concentration of zinc in Znf706 by 4-(2-pyridylazo) resorcinol (PAR) assay. Zinc coordinated cysteines in Znf706 are reduced by para-chloromercuribenzoic acid (PCMB) and compared with Znf706 under a non-reducing condition, without PCMB (-PCMB).

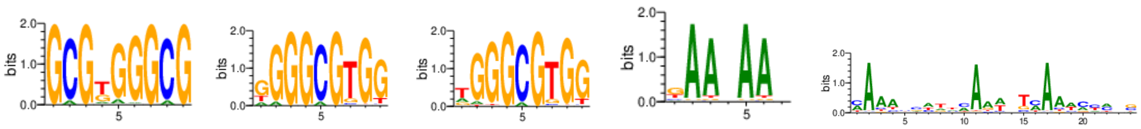


**(a)** **(b)** **(c)** **(d)** **(e)**

**Fig. S2**. Prediction of five C2H2 type zinc-finger proteins that are known to bind to G-rich sequence motifs that include (a) Zif268, (b) Sp-1, and (c) BKLF, yeast AZF1 which is known to bind AAAAGAAA (d), and SALL4 that binds to AT-rich motifs (e) using the Interactive PWM predictor.[10-13]

**a**

| | |
|---|---|
| PolyA | AAAAAAAAAAAAAAAAAAAA |
| PolyN | NNNNNNNNNNNNNNNNNNNN |
| PolyG | GGGGGGGGGGGGGGGGGGGG |
| PolyC | CCCCCCCCCCCCCCCCCCCC |
| PolyT | TTTTTTTTTTTTTTTTTTTT |
| cMyc | TAGGGAGGGTAGGGAGGGT |
| G4C2 | GGGGCCGGGGCCGGGGCCGGGGCC |
| C4G2 | CCCCGGCCCCGGCCCCGGCCCCGG |
| 21-RNA | ACUGCUAGAGAUUUUCCACAU |
| TAR RNA | GGCAGAUCUGAGCCUGGGAGCUCUCUGCC |

**b**

| | |
|---|---|
| O1 | CCGGGTCGGGTGGCC |
| O2 | GGCCCAGCCCACCGG |
| O3 | GGGCTGTGGGTCCCG |
| O4 | CCCGACACCCAGGGC |
| O5 | CCGGTGGGCCGGGAT |
| O6 | GGCCACCCGGCCCTA |
| O7 | GACCGGGCCGTAAGG |
| O8 | CTGGCCCGGCATTCC |
| O9 | GGTGAGGGCGAATTC |
| O10 | CCACTCCCGCTTAAG |
| O11 | GGGGTGGCGCTGGCC |
| O12 | CCCCACCGCGACCGG |
| O13 | TCCGGCTGGGGCCAA |
| O14 | AGGCCGACCCCGGTT |
| O15 | CGCCACCCGTTCCCC |

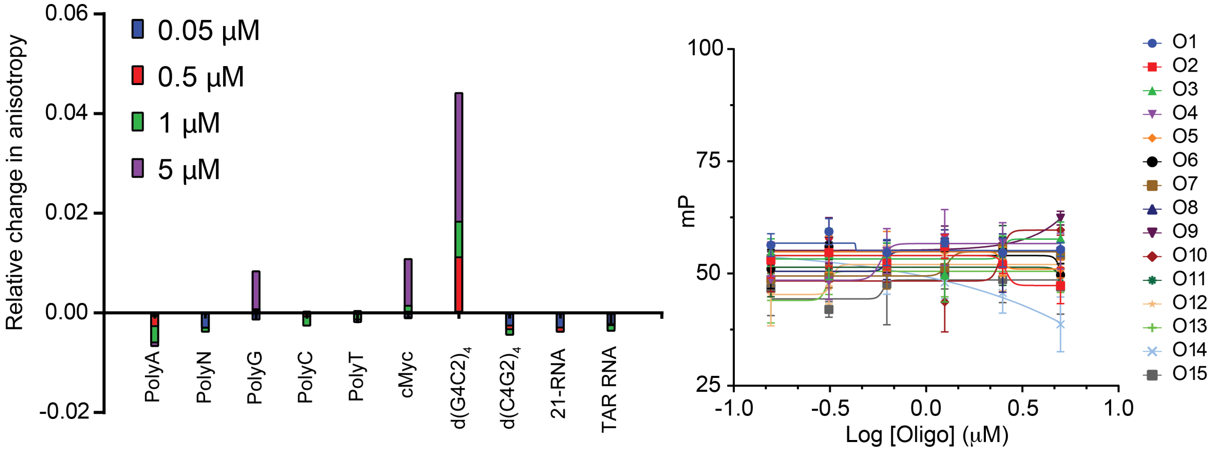


**Fig. S3**. **(a)** Fluorescence anisotropy analysis, shown in lower panel was used to screen the binding specificity of AF488-Znf706 to a number of nucleotide sequences shown in the top panel that include DNA polynucleotides, DNA quadruplexes (cMyc and G4C2), a DNA C4G2 repeat, and single-stranded RNAs (21-mer and TAR) at the oligo concentration that are indicated in a color-coded fashion shown in the lower panel. The only sequences showing significant binding in this assay are PolyG, cMyc and 4-repeat DNA G4C2, all of which are known to form G-quadruplexes. **(b)** Single stranded DNA oligonucleotides named O1-O15 were designed with varying G-repeat sequences for a fluorescence polarization (FP) analysis that is shown in the lower panel. This assay shows changes in polarization value (mP) for 20nM AF-488 Znf706 titrated with a variable concentration of DNA oligonucleotides O1-O15 at the indicated colors. Though G-rich, none of the sequences are predicted to form G-quadruplexes and none appear to bind tightly to Znf706 using this assay.

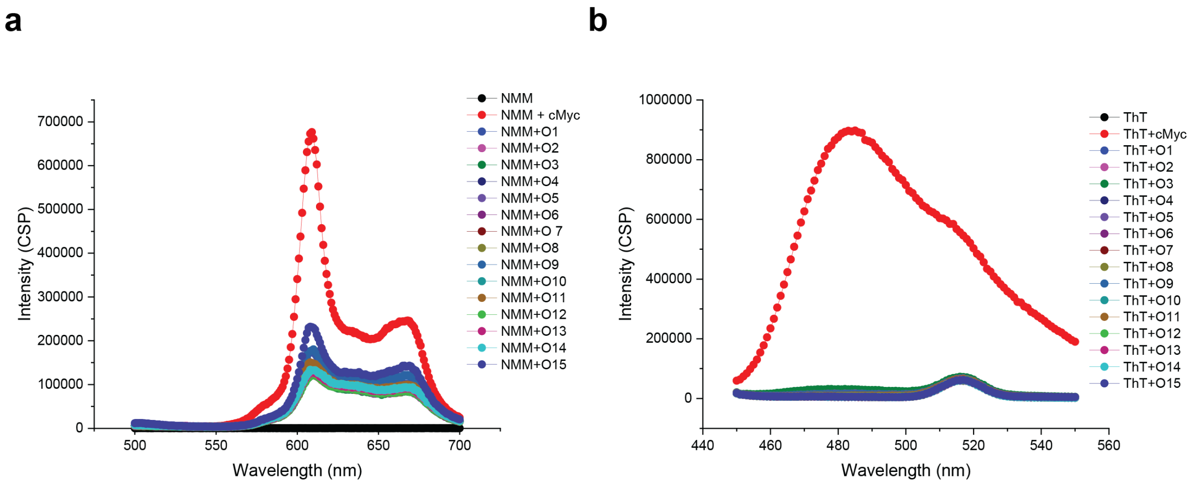


**Fig. S4**. Monitoring the change in 10nM N-methylmesoporphyrin IX (NMM, **a**) and thioflavin-T (ThT, **b**) fluorescence that occurs upon mixing a 10x molar excess of G-rich oligonucleotides O1-O15 or the cMyc G-quadruplex shown as red filled circles (refer to sequence information in **Figs. S3a and b**).

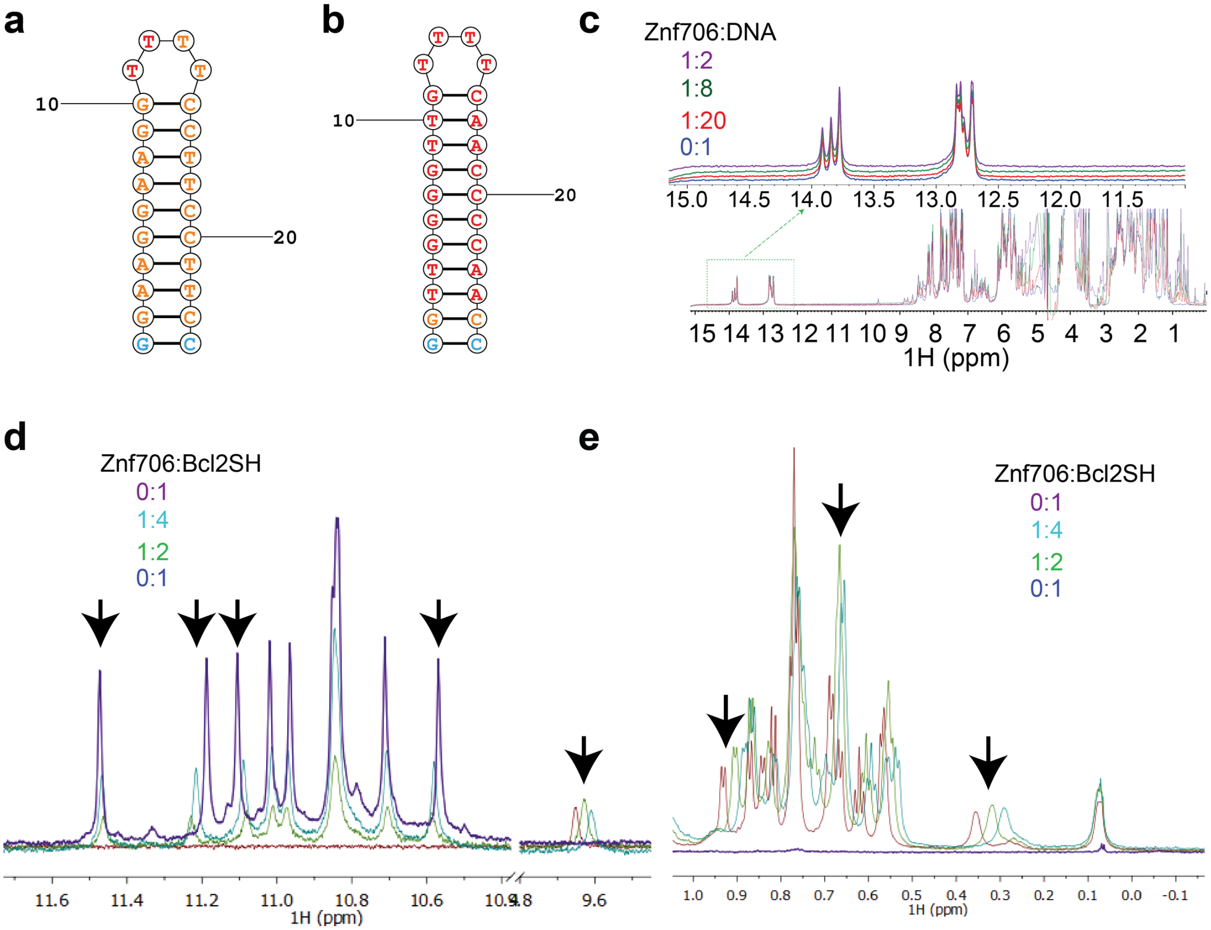


**Fig. S5**. **(a-b)** Secondary structure prediction of GGA **(a)** and GGGG **(b)** hairpin DNA sequences. **(c)** 1D proton NMR spectra of 200 μM GGGG-hairpin oligonucleotide, recorded at 37 °C, titrated with variable concentrations of Znf706 as indicated in the designated colors. Zoomed spectra are shown to highlight that no chemical shift change occurs for the imino protons. A corresponding 1D spectrum of GGA showing no Znf706 induced chemical shifts in imino protons is shown in main text Fig. 1d. **(d-e)** 1D NMR spectrum of Bcl2SH G-quadruplex (200 μM) in the absence or presence of increasing concentrations of Znf706 as shown in the indicated colors. The imino **(d)** and methyl **(e)** protons showing chemical shift perturbations upon Znf706 addition are indicated with arrows. The 1D proton NMR spectrum of Znf706 in the absence of DNA is presented in maroon color.

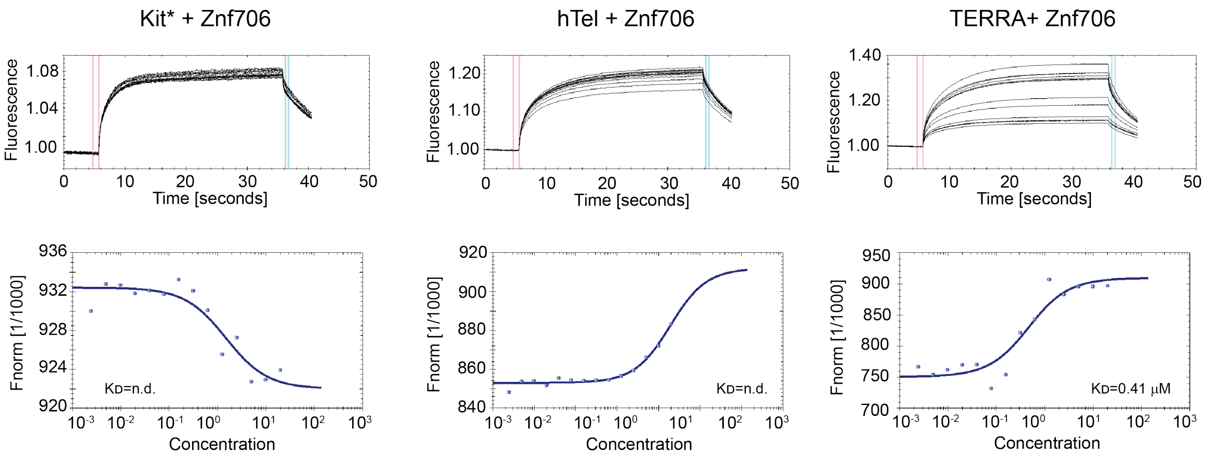


**Fig. S6**. Microscale thermophoresis of the interaction between Znf706 and G-quadruplexes. MST traces of a titration of Znf706 against 100 nM DNA (hTel and Kit*) and RNA (TERRA: UA GGG UUA GGG UUA GGG UUA GGG) 6-FAM G-quadruplexes. Traces on the top and bottom represent bound and unbound fractions, respectively. The MST curves are analyzed using the Nanotemper analysis program and the dissociation constant ($K_D$) as shown in figure for each system was calculated using the equation $f(c) = unbound + \frac{(bound - unbound)}{2} \times (FluoConc + c + Kd - \sqrt{((FluoConc + c + Kd)^2 - 4 \times FluoConc \times c)}$.

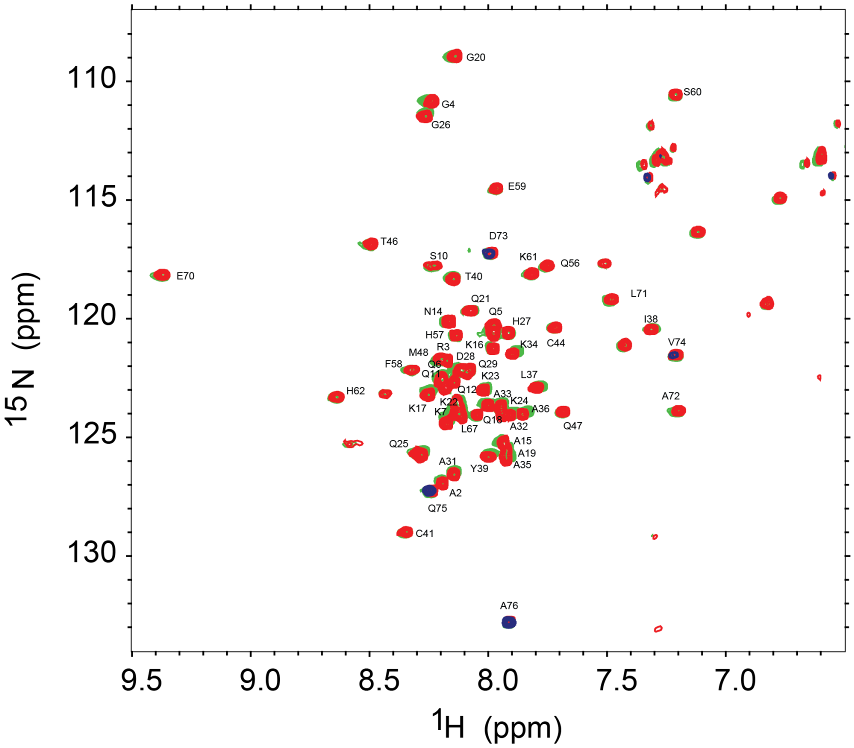


**Fig. S7**. TROSY spectrum of 50 μM $^{15}N$ Znf706 (red spectrum) mixed with 100 μM $(G4C2)_4$ prepared in 20 mM NaPi, 0.05% $NaN_3$, 7.5% $D_2O$, pH 7.4, 100 mM KCl (blue spectrum) or in 20 mM NaPi, 7.5% $D_2O$, 0.05% $NaN_3$, pH 7.4, 100 mM NaCl (green spectrum). The TROSY spectra were acquired at 4 °C on a Bruker 800 MHz spectrometer.

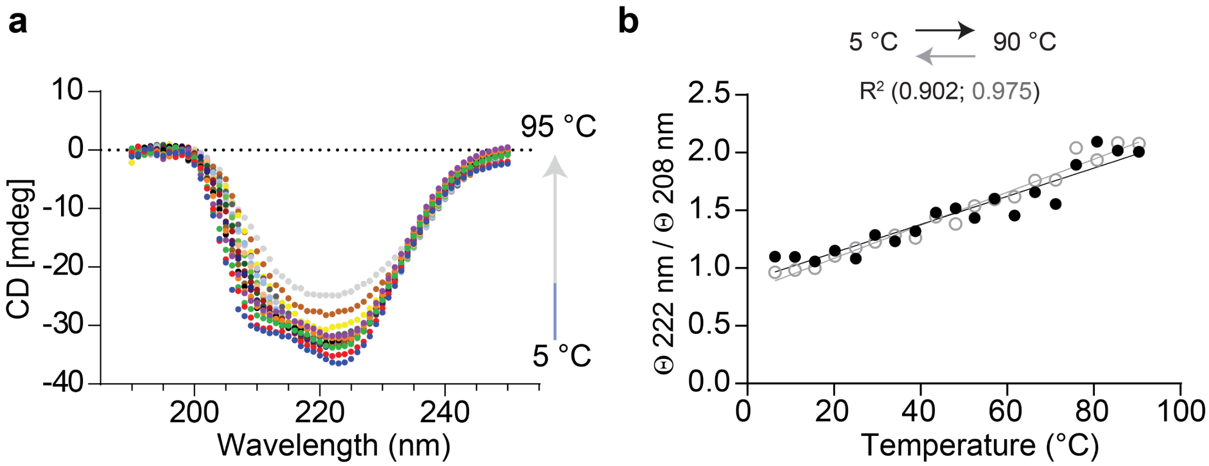


**Fig. S8**. **(a)** Monitoring Znf706 (50 μM) conformational changes as a function of temperature by CD spectroscopy. CD spectra were recorded in every 5 °C interval in temperatures ranging from 5 to 95 °C. **(b)** Monitoring the conformational reversibility in 50 μM Znf706 by CD spectroscopy as a function of the change in CD signal intensity ratio at 222 and 208 nm. The folding and unfolding are reversible. However, the direct correlation between temperature and Znf706 secondary structure suggests a highly noncooperative folding transition. Solid and open circles represent ellipticity recorded during the melting (5 to 90 °C) and cooling cycle (90 to 5 °C), respectively.

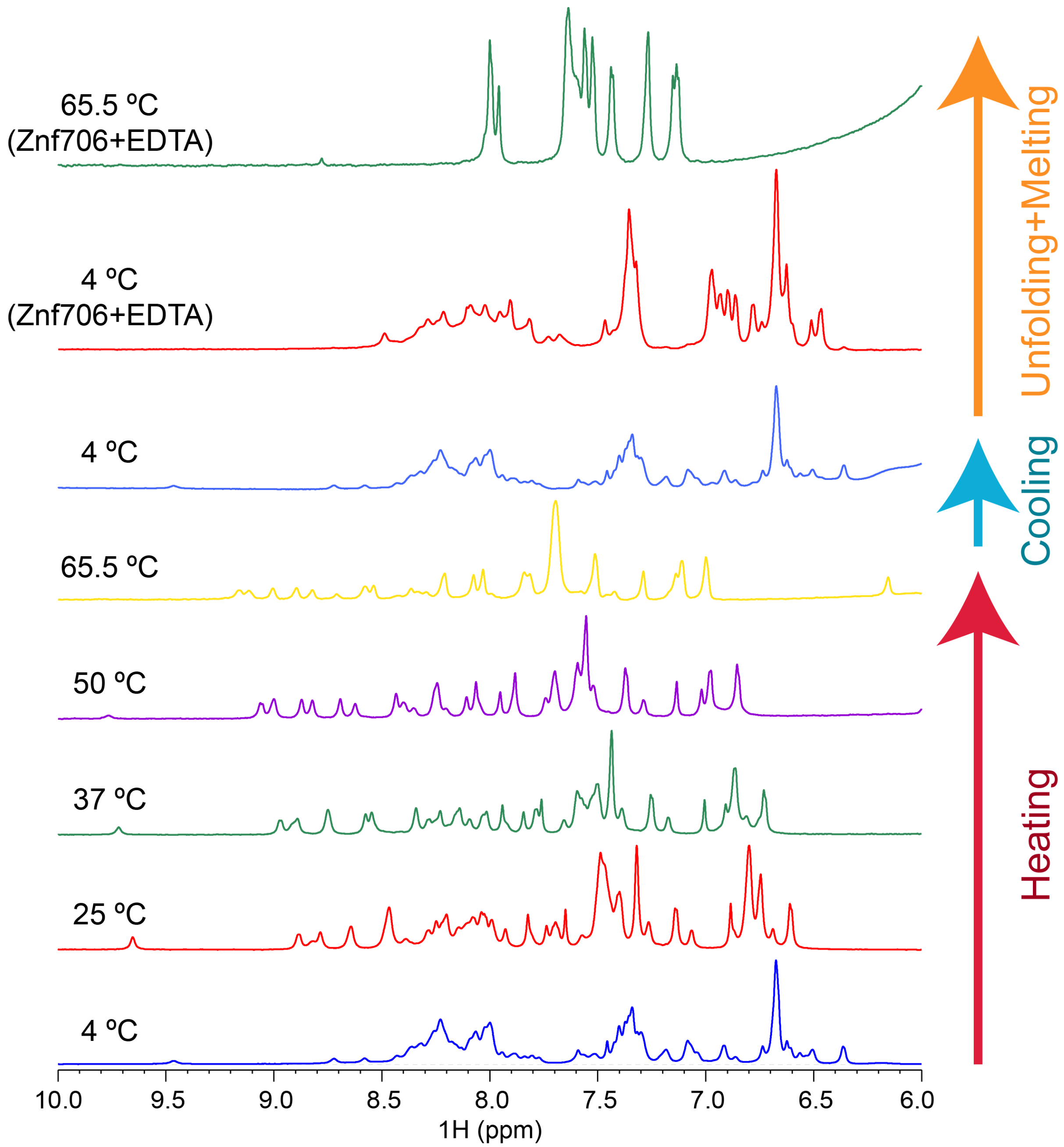


**Fig. S9**. Monitoring thermostability of Znf706 using 1D proton NMR. Proton NMR spectra of 100 µM Znf706 dissolved in 20 mM NaPi, 100 mM KCl, 0.05% $NaN_3$, pH 7.4 were recorded at the indicated temperatures. Znf706 was slowly heated from 4 °C to 65.5 °C (red arrow) and then cooled back to 4 °C (blue arrow) followed by 10-molar excess EDTA treatment to test its effect on Znf706 structure (orange arrow). 1D NMR spectra were recorded on a Bruker 800 MHz spectrometer.

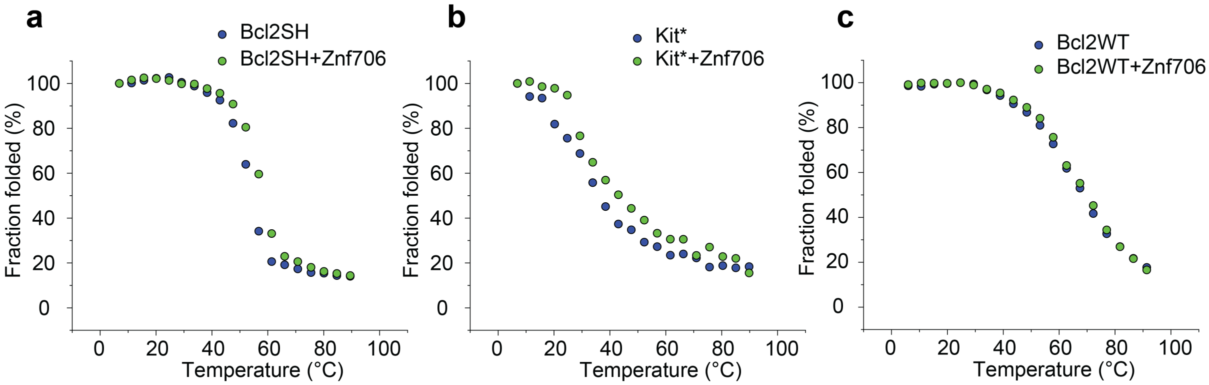


**Fig. S10**. CD melting temperature analysis of 20 μM Bcl2SH **(a)**, Kit* **(b)**, and Bcl2WT **(c)** in the absence (blue) or presence of equimolar Znf706 (green).

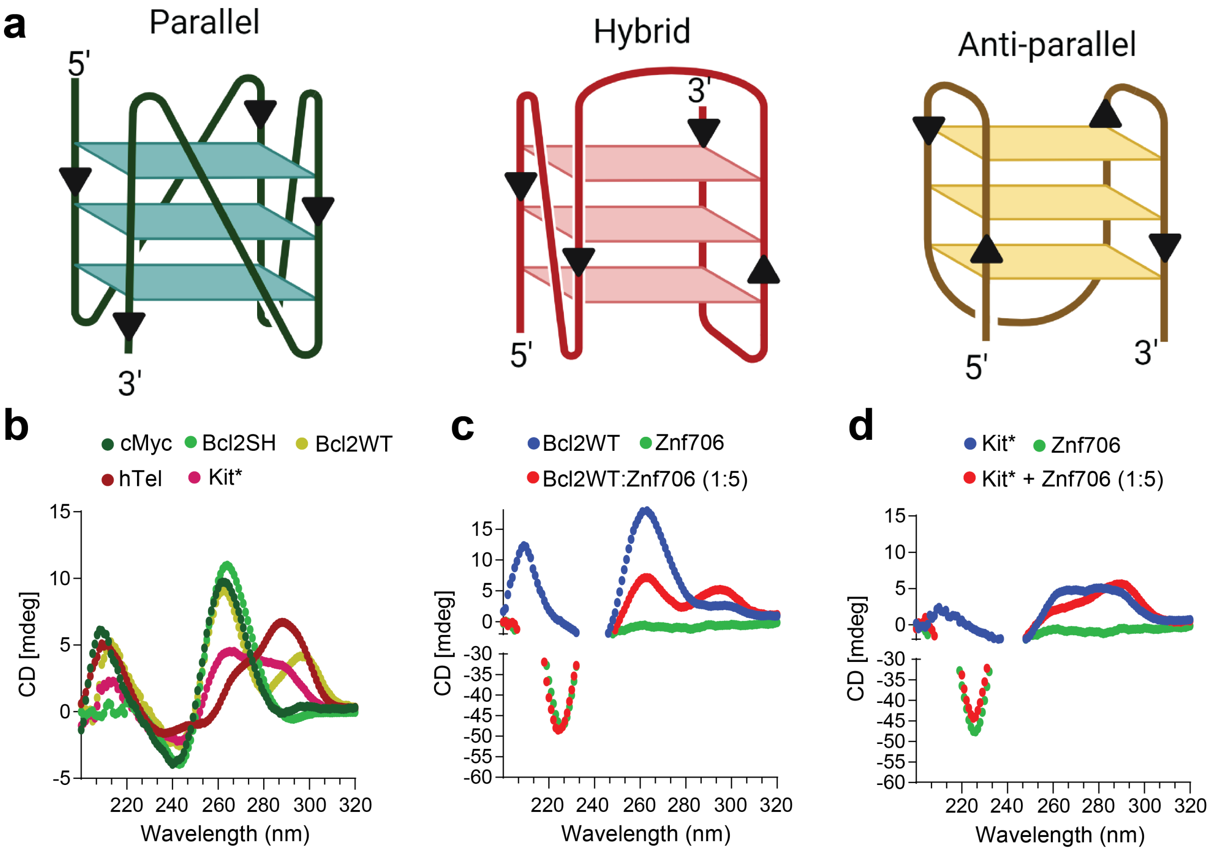


**Fig. S11**. **(a)** Schematic representation of three different G-quadruplex topologies. **(b)** CD spectrum of 20 µM G-quadruplexes is characterized with three different topologies. CD spectrum for cMyc and Bcl2SH is typical for quadruplexes with parallel topology; hybrid topology is indicated for hTel, Kit* and BCl2WT. **(c-d)** Effect of Znf706 on the refolding of Bcl2WT **(c)** and Kit* (**d**) as studied using CD spectroscopy. Addition of a 5x molar excess Znf706 induces antiparallel conformation of 20 µM Bcl2WT and Kit* prepared in 20 mM NaPi, 4 mM KCl, pH 7.4.

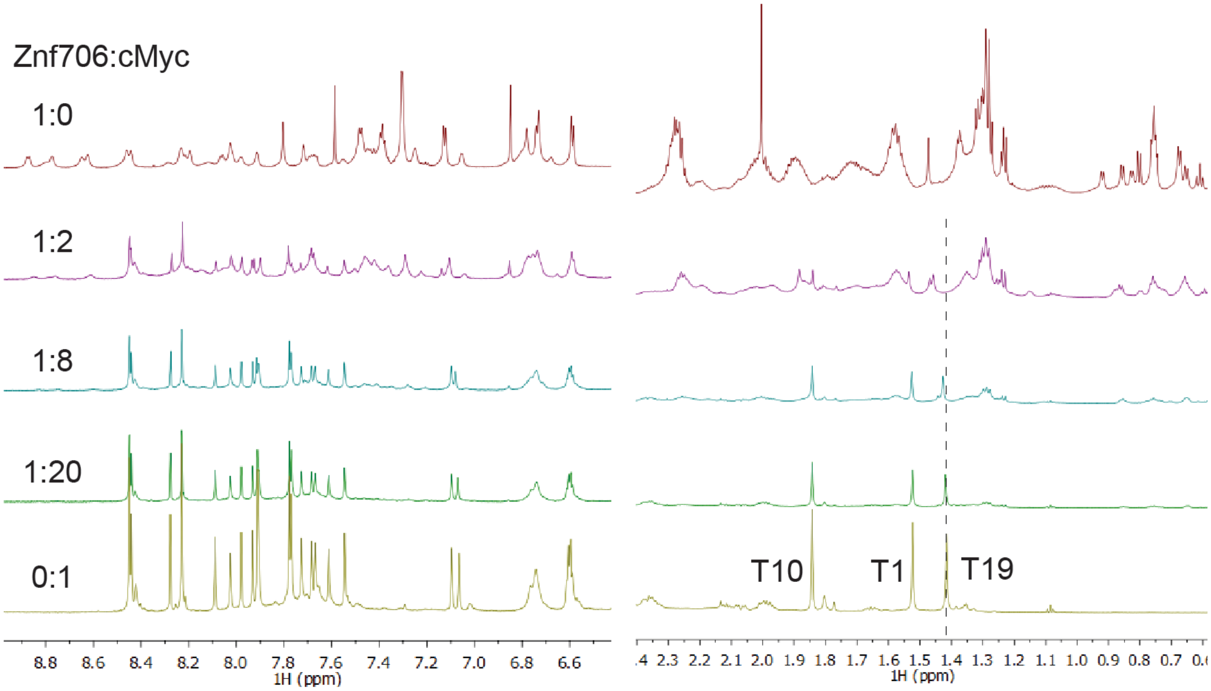


**Fig. S12**. 1D NMR spectra (aromatic regions: left, methyl regions: right) of 200 μM cMyc mixed with an increasing concentrations of Znf706. The methyl peaks are labelled, and the dashed line indicates a chemical shift change in T19 that occurs upon Znf706 binding.

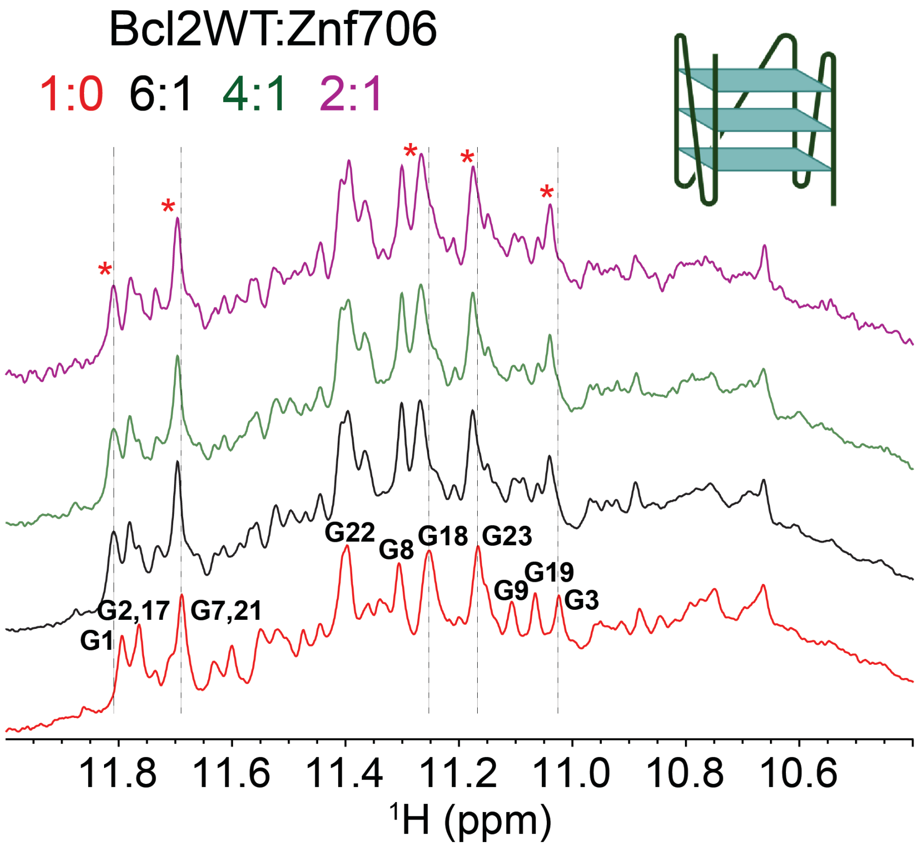


**Fig. S13**. Mapping of the specific interaction sites within the Bcl2WT structure with which Znf706 binds using 1D proton NMR. The guanines in Bcl2WT that have assigned chemical shifts[14] are labelled on the red spectrum. When increasing concentrations of Znf706 is added, several Bcl2WT G-quadruplex imino protons show chemical shift changes as highlighted by the dashed lines. These represent guanines directly involved in Znf706 interaction and are denoted with a red asterisk "*".

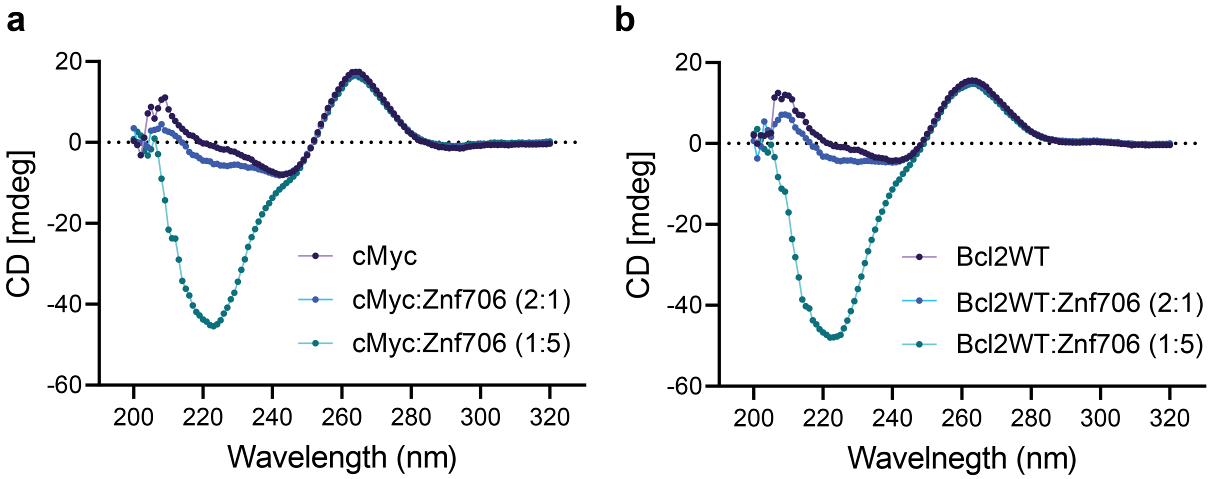


**Fig. S14**. CD spectroscopy analysis to probe the binding effect of Znf706 binding on cMyc **(a)** and Blc2WT **(b)** G-quadruplex secondary structures. 20 µM G-quadruplexes were folded in 20 mM NaPi, 100 mM KCl, pH 7.4 and the effect of Znf706 addition dissolved in an identical buffer was monitored at 25 °C at the indicated molar ratios.

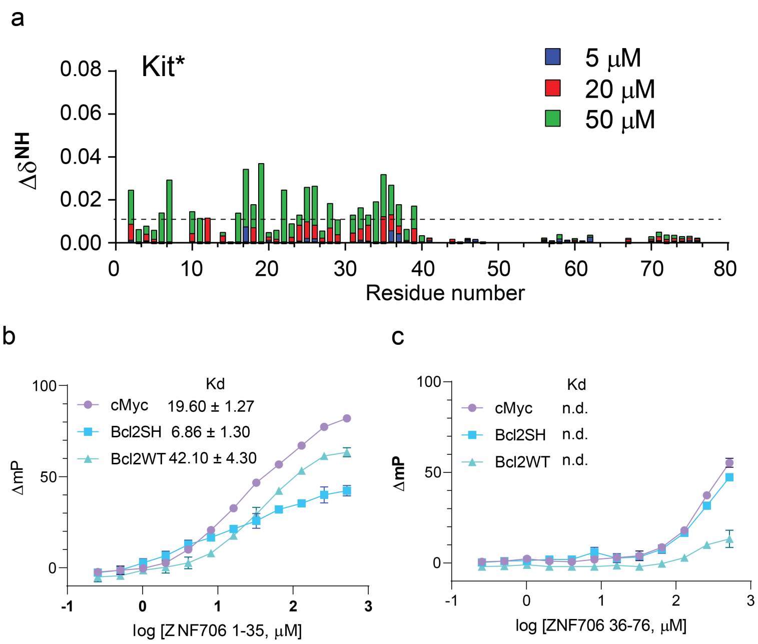


**Fig. S15**. **(a)** Plot showing chemical shift perturbations (CSPs) derived from the $^{15}N/^{1}H$ 2D spectrum of 100 μM Znf706 titrated with variable concentrations of Kit* as indicated in colors. The CSPs are calculated using equation $\Delta\delta_{NH} = \sqrt{(\delta^{1}H)^{2} + 0.154 \times (\delta^{15}N)^{2}}$ and the dashed lines indicate the average CSPs. Binding titrations of Znf706 N-terminal residues 1-35 **(b)** and C-terminal zinc-finger residues 36-76 **(c)** and FAM-labeled G-quadruplexes prepared in 20 mM NaPi, 100 mM KCl, pH 7.4. The indicated $K_d$ values were calculated by non-linear regression analysis using a one site binding saturation model in GraphPad Prism at increasing concentrations of protein (0.25 μM to 520 μM). Error bars represent s.d derived from three replicates. n.d.: not determined.

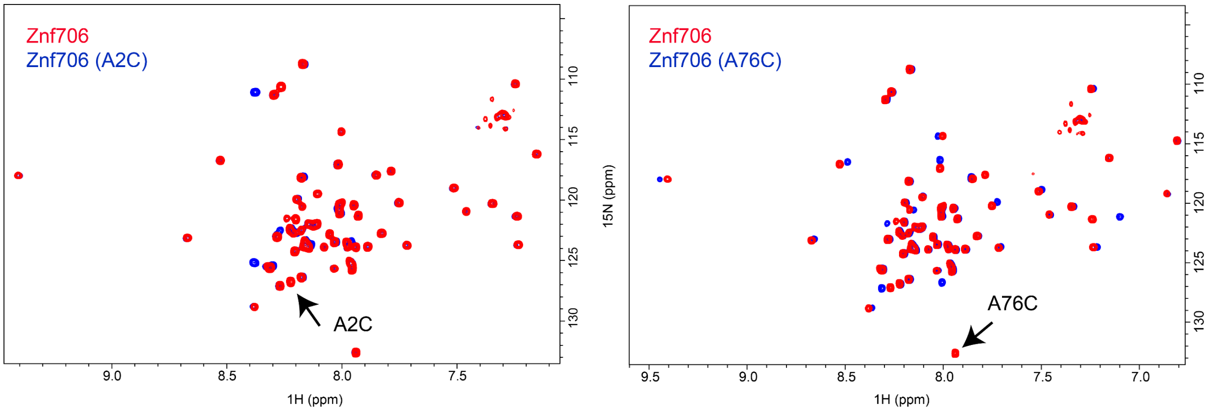


**Fig. S16**. Overlay TROSY spectrum of 100 μM $^{15}N$ labelled Znf706 in red and Znf706 A2C (left) or A76C (right) in blue prepared in 20 mM NaPi, 100 mM KCl, 0.05% $NaN_3$, pH 7.4 containing 7.5% $D_2O$ recorded on a Bruker 800 MHz at 4°C.

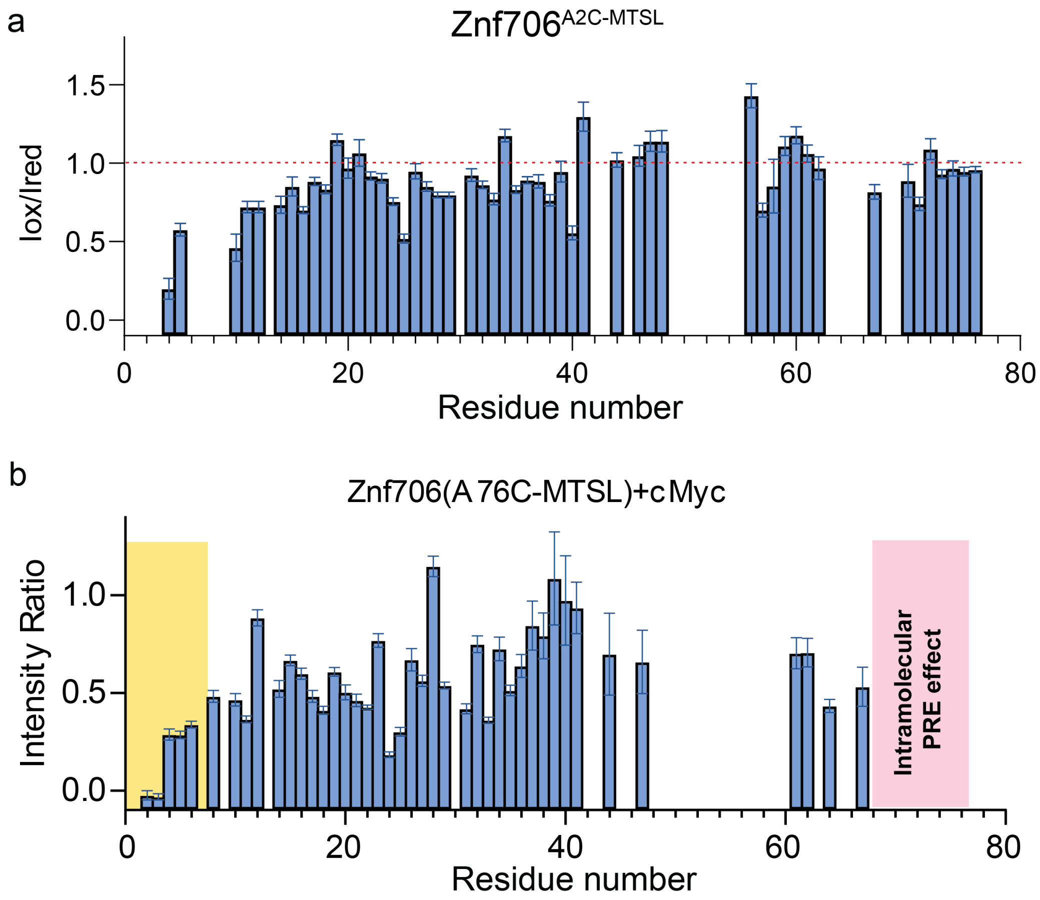


**Fig. S17**. **(a)** Intensity ratio of amide protons observed for 100 µM $^{15}$N Znf706 A2C-MTSL mixed with or without 50 µM cMyc G-quadruplex as indicated in oxidized (paramagnetic state) and reduced (diamagnetic state) conditions. The Znf706 A2C-MTSL NMR sample was reduced with 5-molar excess sodium-ascorbate for ~3 hour prior to recording the diamagnetic spectrum. The NMR data were recorded on a Bruker 800 MHz spectrometer at 4 °C for samples dissolved in 20 mM NaPi, 0.05% $NaN_3$, 100 mM KCl, pH 7.4 buffer containing 7.5% $D_2O$. **(b)** Signal intensity ratio of amide protons observed for 100 µM $^{15}$N Znf706 A76C-MTSL mixed with 50 µM cMyc G-quadruplex. Standard errors are estimated from the signal-to-noise ratios. The intramolecular PRE effects at C-terminus are shaded in pink, whereas cMyc binding induced PRE effects are highlighted in yellow. NMR spectra were collected on a Bruker 800 MHz spectrometer at 4 °C for samples dissolved in 20 mM NaPi, 100 mM KCl, pH 7.4 buffer containing 7.5% $D_2O$.

Znf706 + cMyc

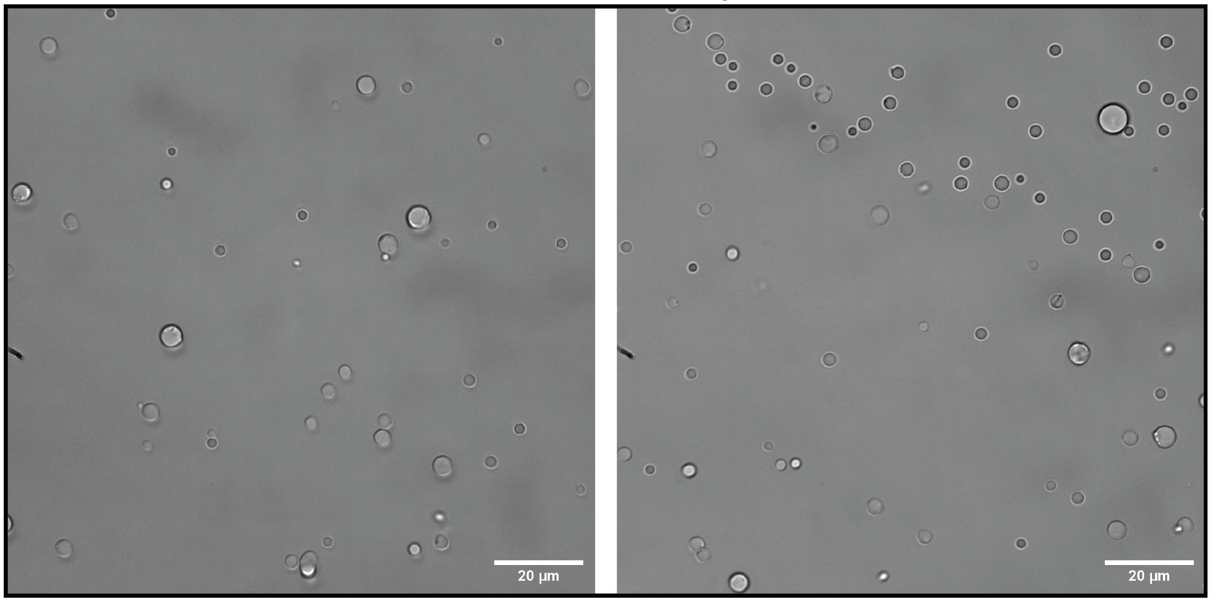


**Fig. S18**. Differential interference contrast (DIC) images showing the formation of droplets in sample mixture containing 100 µM Znf706 and 100 µM cMyc incubated overnight at room temperature. The sample mixture was dissolved in 20 mM NaPi, 100 mM KCl, pH 7.4 and 7.5% $D_2O$ inside a 0.5 mL NMR tube and transferred to a 16-well CultureWell™ prior to imaging.

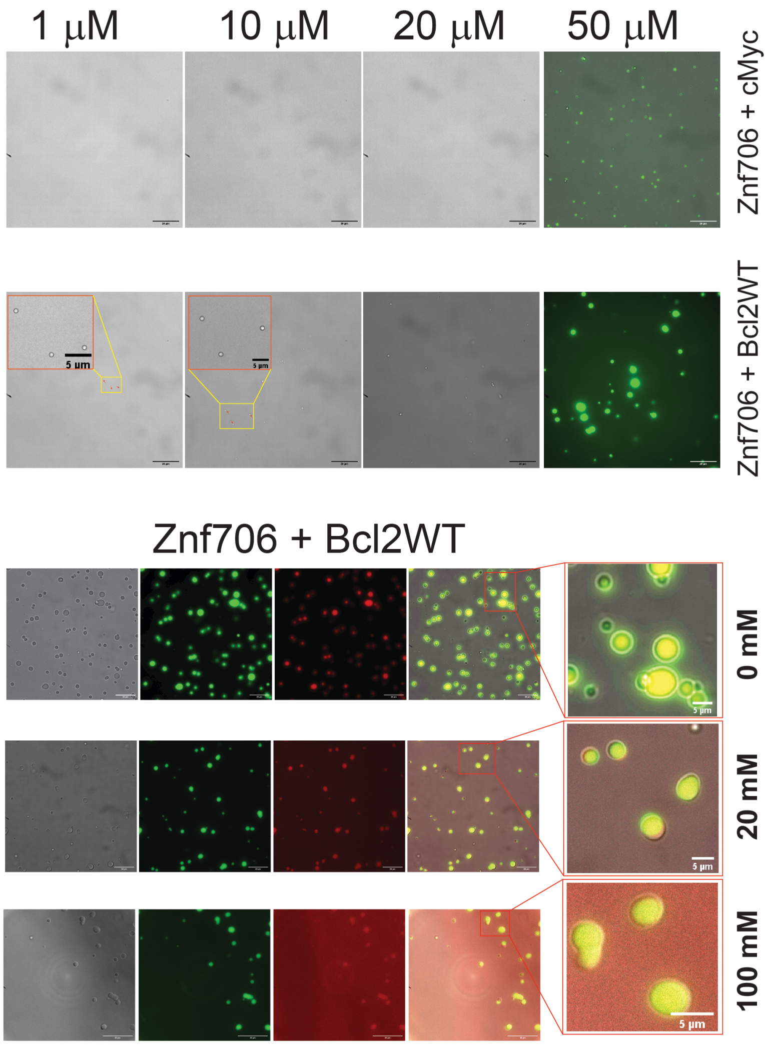


**Fig. S19**. Fluorescence imaging to test the formation of LLPT in Znf706 at different concentrations (1 μM to 50 μM) mixed with an equimolar amount of cMyc or Bcl2WT (top). Monitoring the formation of LLPT in a sample mixture containing 100 μM Znf706 and an equimolar amount of Bcl2WT dissolved in 20 mM NaPi, pH 7.4 at the indicated KCl concentrations (bottom). For fluorescence imaging, a sample mixture ratio of 1:100 AF488-Znf706:unlabeled-Znf706 was used. The samples were incubated overnight at room temperature prior to imaging. LLPT formation was confirmed by DIC following addition of 5 μM NMM to the sample mixture. The green and red signals correspond to AF488-Znf706 and NMM, respectively.

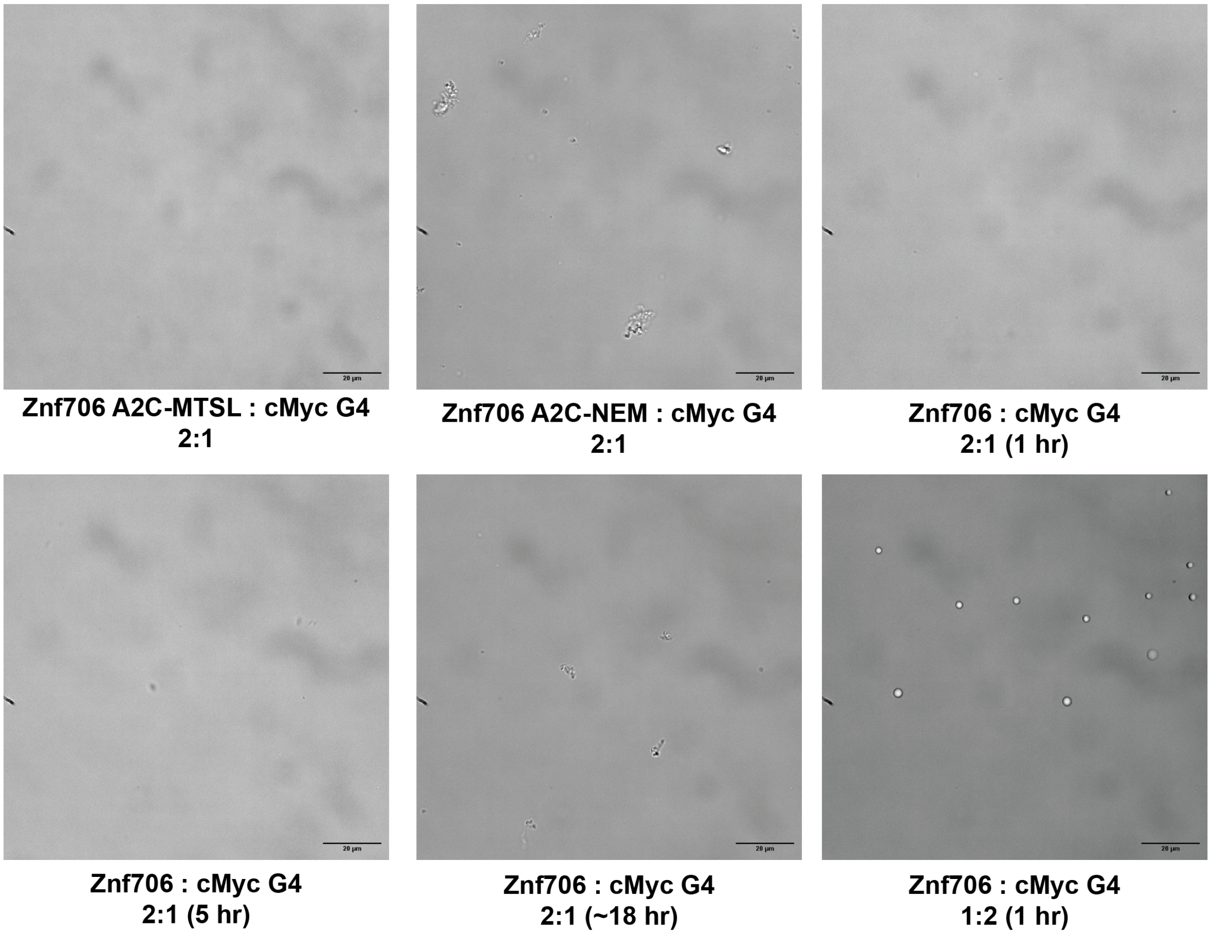


**Fig. S20**. Monitoring liquid-liquid phase transition in Znf706 (100 µM) or Znf706-A2C (100 µM) labelled with MTSL, or NEM incubated with 50 µM of cMyc G4 at room temperature at the indicated timepoints (top panel). The samples were dissolved in 20 mM NaPi, 100mM KCl, pH 7.4 and 7.5% $D_2O$ corresponding to the NMR samples (protein:G4=2:1) used for 2D and PRE NMR measurements. Scale bar is 20 µm. Liquid droplets were observed in sample mixtures containing 50 µM Znf706 and 100 µM cMyc as indicated within ~1 hour of incubation at room temperature.

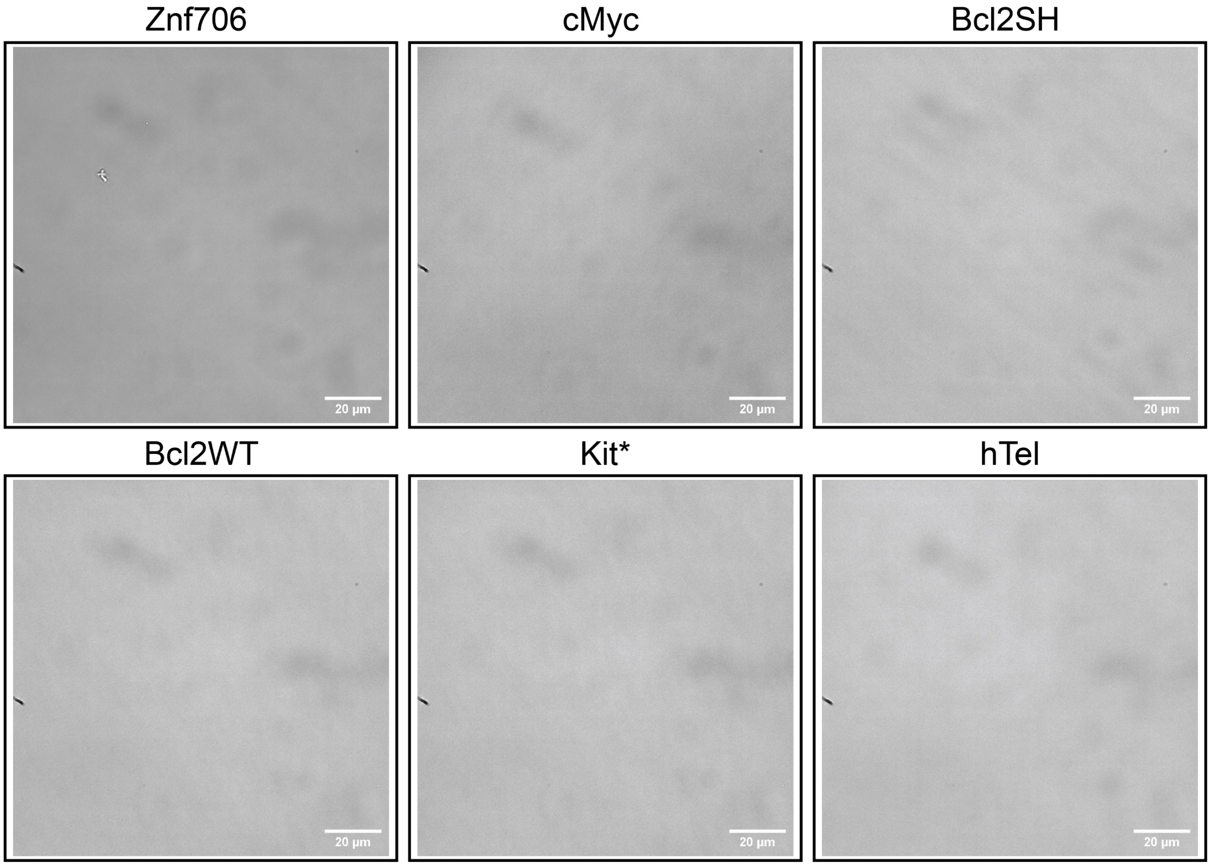


**Fig. S21**. Differential interference contrast (DIC) images showing no LLPT formed when 100 µM Znf706 or G-quadruplexes alone were dissolved in 20 mM NaPi, 100 mM KCl, 7.5% $D_2O$, pH 7.4 and incubated overnight at room temperature prior to imaging.

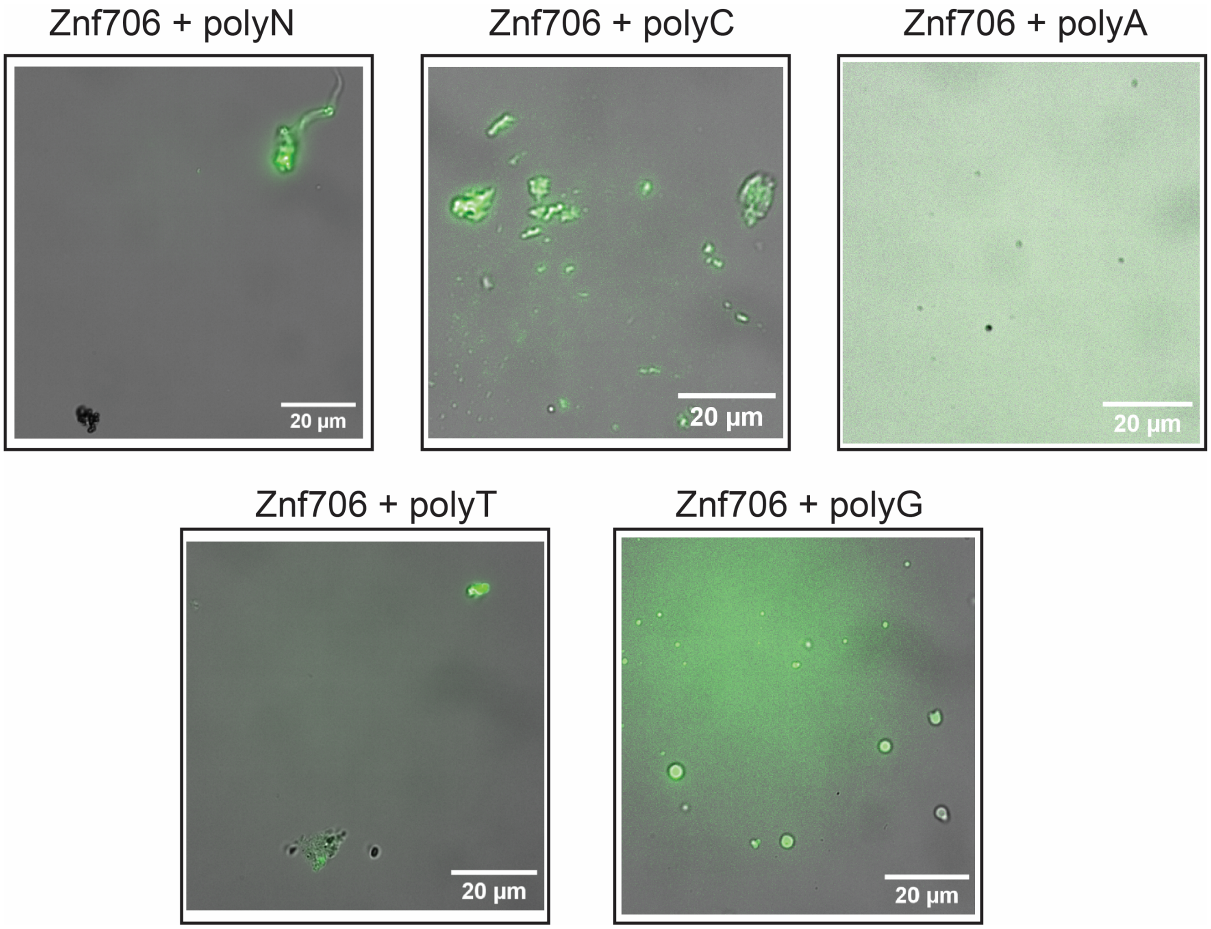


**Fig. S22**. Fluorescence imaging to test the formation of LLPT for a sample mixture containing 100 µM Znf706 and equimolar DNA polynucleotides dissolved in 20 mM NaPi, 100 mM KCl, 0.05% $NaN_3$, 7.5% $D_2O$, pH 7.4. The samples were incubated overnight at room temperature prior to imaging.

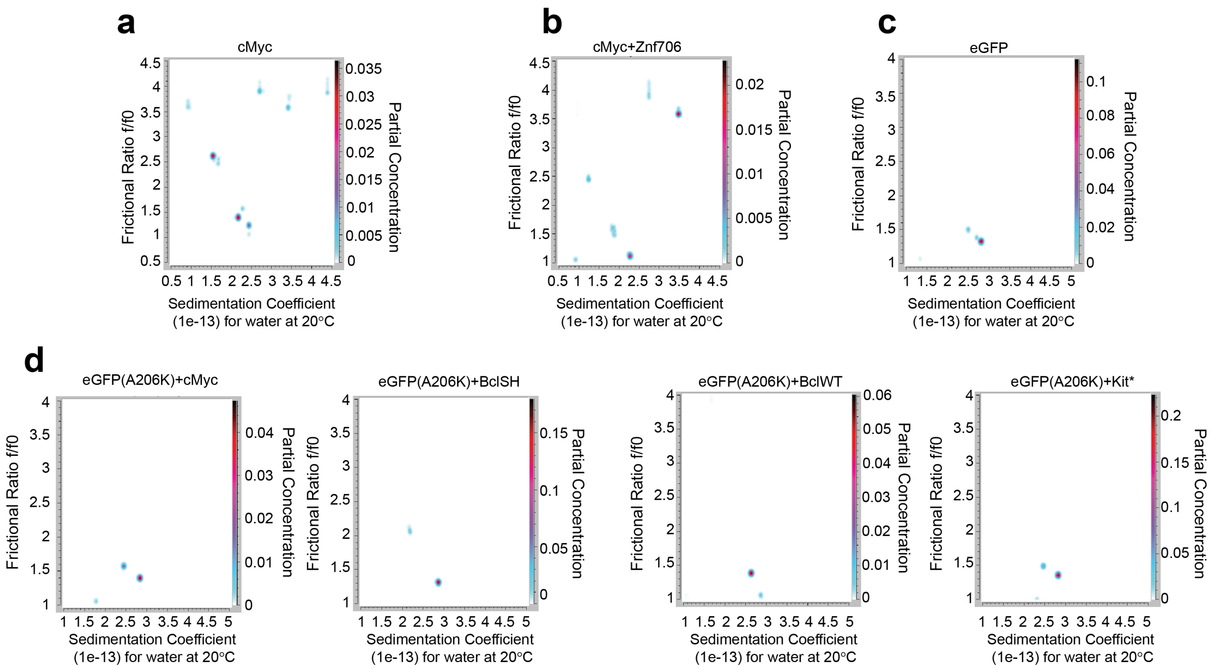


**Fig. S23**. Determination of Znf706 and G-quadruplex complex sizes using analytical ultracentrifugation (AUC). cMyc (3.6 μM) dissolved in 20 mM NaPi, 100 mM KCl, pH 7.4 **(a)** was incubated with equimolar Znf706 **(b)** or for 1 hour prior to AUC measurement (absorbance 260 nm). AUC measurement of eGFP (12.2 μM) mixed without **(c)** or with different G-quadruplexes (12.2 μM) as indicated **(d).** Samples were incubated in the dark for ~1 hour and AUC measurements were done recording the absorbance at 488 nm. AUC data are analyzed in Ultrascan and two-dimensional plots are shown. The partial concentration shown in color in the z dimension represents the abundance of each species.

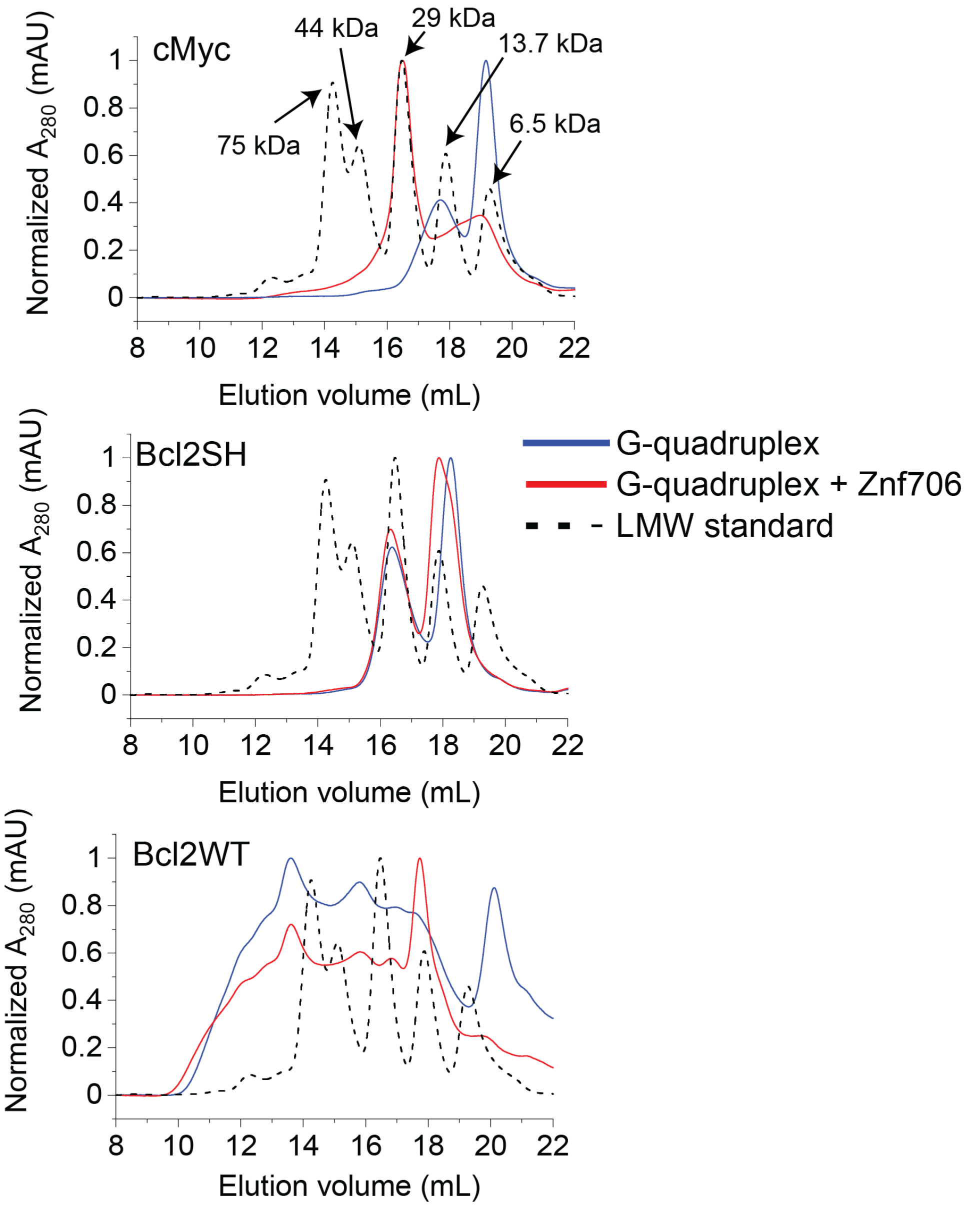


**Fig. S24**. Size-exclusion chromatography profiles of three different G-quadruplexes (2.5 μM) mixed with or without an equimolar amount of Znf706. The SEC profile of a low-molecular weight (LMW) standard is shown in dashed lines. Samples were prepared in 20 mM NaPi, 100 mM KCl, pH 7.4 and incubated ~2 hours prior loading to the SEC column.

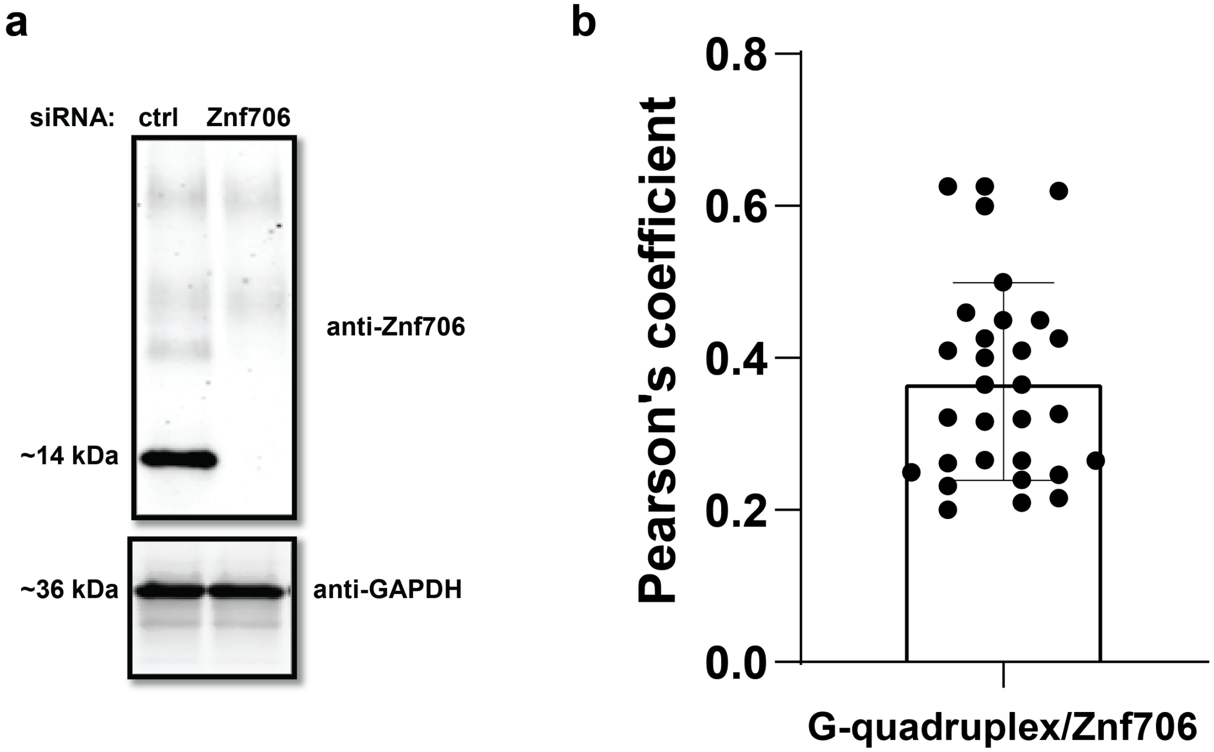


**Fig. S25**. **(a)** Western blot analysis of Znf706 using a polyclonal rabbit anti-Znf706 antibody. **(b)** Pearson's coefficient analysis of colocalized Znf706 and DNA G-quadruplex signals in 30 cells was calculated using ImageJ. The fluorescence images used for the Pearson's analysis are shown in **Fig. 6a**.

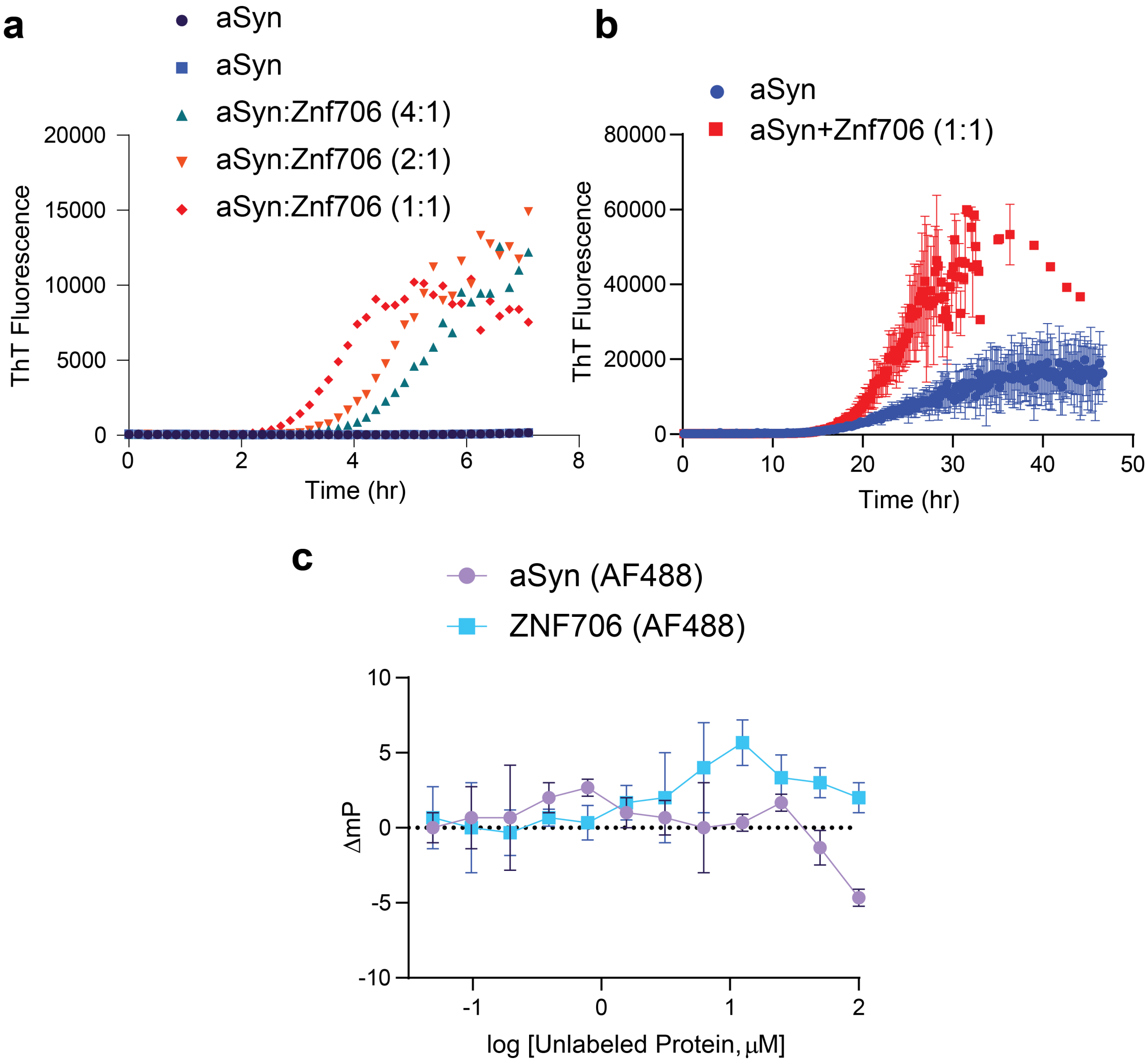


**Fig. S26**. **(a)** Monitoring aggregation kinetics of 100 μM α-Synuclein (aSyn) dissolved in 20 mM NaPi, 100 mM KCl, pH 7.4 mixed with and without Znf706 at different stoichiometries as indicated by the colors analyzed by a thioflavin-T fluorescence assay. **(b)** Aggregation kinetics of 50 μM a-Synuclein mixed without (blue) or with (red) 50 μM Znf706. Standard deviation shown are obtained from four replicates. **(c)** Fluorescence polarization assay showing no interaction between Znf706 and a-Synuclein prepared in 20 mM NaPi, 100 mM KCl, pH 7.4. Fluorescence polarization binding curves are obtained for samples containing 100 nM of AF488 aSyn/Znf706 and variable concentration of Znf706/aSyn incubated at room temperature for ~15 minutes.

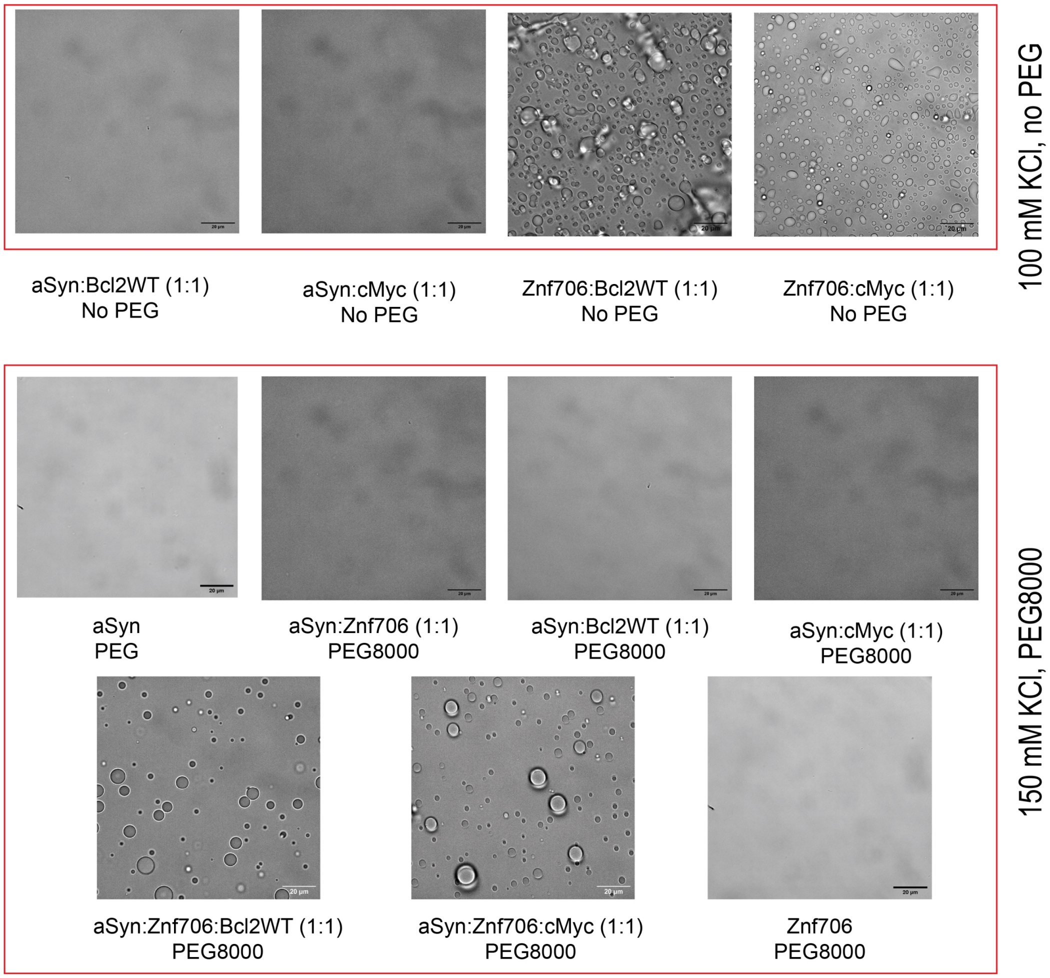


**Fig. S27**. Effect of α-Synuclein (aSyn) on Znf706 and G-quadruplex liquid-liquid phase transition. DIC images showing the droplet formation in 100 μM α-Synuclein mixed with or without an equimolar concentration of Znf706 and/or G-quadruplexes. The droplet formation was tested in 20 mM NaPi sample buffer containing no PEG (100 mM KCl) or 10% PEG8000 (150 mM KCl). Scale bar is 20 μm.

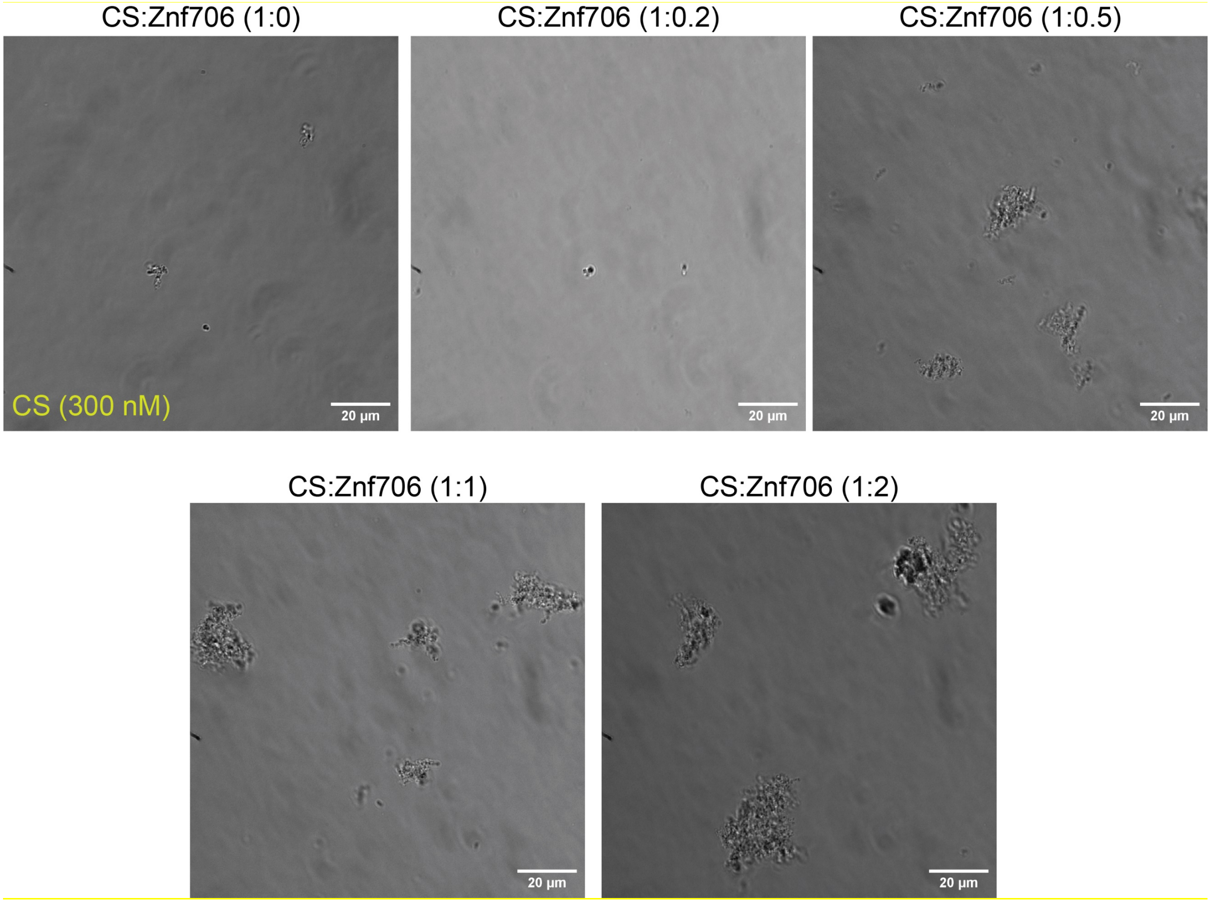


**Fig. S28**. DIC images of citrate synthase (CS) amorphous aggregates obtained from thermal aggregation experiments. Thermal aggregation of 300 nM CS mixed without or with the indicated Znf706 concentrations was done at 48 °C.

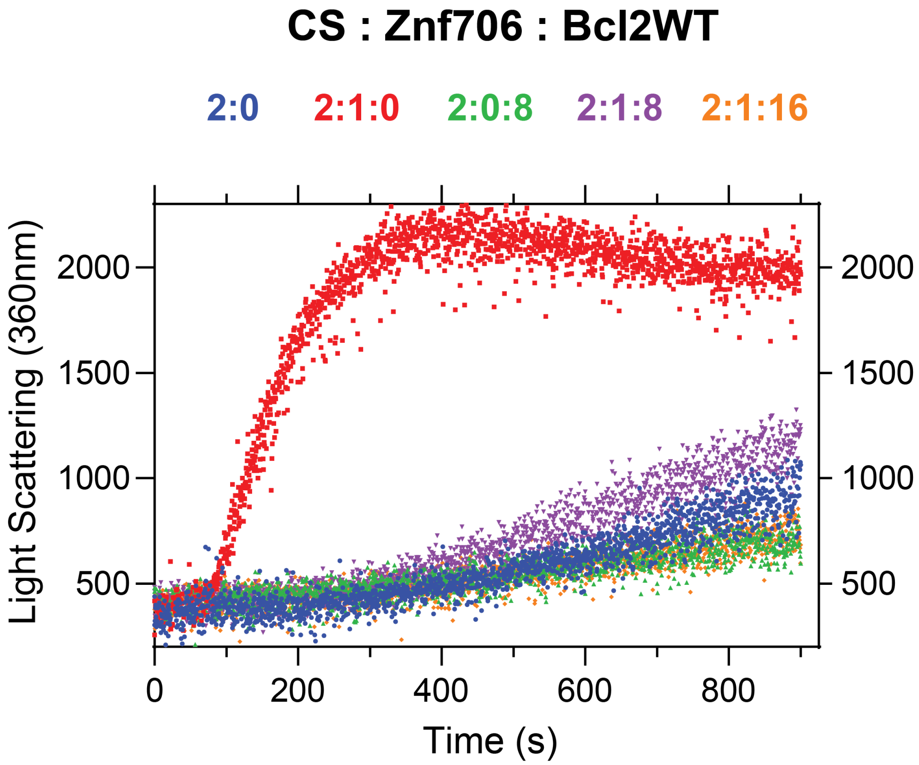


**Fig. S29**. Thermal aggregation curves of 300 nM CS mixed without (blue) or with the indicated Znf706 and Bcl2WT G-quadruplex concentrations. The aggregation kinetics of CS was done at 48 °C in 40 mM HEPES-KOH, pH 7.5.

**Video SV1**. LLPT droplets formed by mixing 100 µM of Bcl2WT G-quadruplex and 100 µM of Znf706. The imaging was done post-incubation of the sample mixture for overnight at room temperature.

**Video SV2**. LLPT droplets formed by mixing 100 µM of cMyc G-quadruplex and 100 µM of Znf706. The imaging was done post-incubation of the sample mixture for overnight at room temperature.

**References**

1. Meinen, B. A., Gadkari, V. V, Stull, F., Ruotolo, B. T. & Bardwell, J. C. A. SERF engages in a fuzzy complex that accelerates primary nucleation of amyloid proteins. **116**, 23040–23049 (2019).
2. Eswar, N. *et al.* Comparative Protein Structure Modeling Using MODELLER. *Curr Protoc Protein Sci* **50**, 2.9.1-2.9.31 (2007).
3. De Vries, S. J., Van Dijk, M. & Bonvin, A. M. J. J. The HADDOCK web server for data-driven biomolecular docking. *Nat Protoc* **5**, 883-97 (2010).
4. Martin M. Cutadapt removes adapter sequences from high-throughput sequencing reads. *EMBnet J* **17**, 10 (2011).
5. Kim, D., Langmead, B. & Salzberg, S. L. HISAT: A fast spliced aligner with low memory requirements. *Nat Methods* **12**, 357-360 (2015).

6. Pertea, M. et al. StringTie enables improved reconstruction of a transcriptome from RNA-seq reads. *Nat Biotechnol* **33**, 290-295 (2015).
7. Love, M. I., Huber, W. & Anders, S. Moderated estimation of fold change and dispersion for RNA-seq data with DESeq2. *Genome Biol* **15**, 550 (2014).
8. Chambers, V. S. et al. High-throughput sequencing of DNA G-quadruplex structures in the human genome. *Nat Biotechnol* **33**, 877-881 (2015).
9. Begeman, A. *et al.* G-Quadruplexes act as sequence-dependent protein chaperones. *EMBO Rep* **21**, e49735 (2020).
10. Kong, N. R. *et al.* Zinc Finger Protein SALL4 Functions through an AT-Rich Motif to Regulate Gene Expression. *Cell Rep* **34**, (2021).
11. Iuchi, S. Three classes of C2H2 zinc finger proteins. *Cellular and Molecular Life Sciences* **58,** 625-635 (2001).
12. Persikov, A. V. & Singh, M. De novo prediction of DNA-binding specificities for Cys2His 2 zinc finger proteins. *Nucleic Acids Res* **42**, 97–108 (2014).
13. Slattery, M., Liko, D., Heideman, W. The function and properties of the Azf1 transcriptional regulator change with growth conditions in *Saccharomyces cerevisiae. Eukaryotic Cell* **5,** 313–320 (2006).
14. Dai, J. *et al.* An intramolecular G-quadruplex structure with mixed parallel/antiparallel G-strands formed in the human BCL-2 promoter region in solution. *J Am Chem Soc* **128**, (2006).
